# Reference Electrode Types for Zero‐Gap CO_2_ Electrolyzers: Benefits and Limitations

**DOI:** 10.1002/advs.202402095

**Published:** 2024-06-25

**Authors:** Luca Bohn, Jochen Kieninger, Stefan J. Rupitsch, Carolin Klose, Severin Vierrath, Joey Disch

**Affiliations:** ^1^ Electrochemical Energy Systems, IMTEK – Department of Microsystems Engineering University of Freiburg Georges‐Koehler‐Allee 103 79110 Freiburg Germany; ^2^ FIT – Freiburg Center for Interactive Materials and Bioinspired Technologies University of Freiburg Georges‐Koehler‐Allee 105 79110 Freiburg Germany; ^3^ Laboratory for Electrical Instrumentation and Embedded Systems, IMTEK – Department of Microsystems Engineering University of Freiburg Georges‐Koehler‐Allee 106 79110 Freiburg Germany

**Keywords:** CO_2_ electrolysis, electrochemical CO_2_ reduction, electrode potential, reference electrode integration, zero‐gap cell

## Abstract

Integrated reference electrodes allow to deconvolute voltage contributions of anode and cathode and contribute to a better understanding of CO_2_ electrolyzers. However, in zero‐gap cell configurations, this integration can be challenging and obtaining error‐free data with such a setup is a non‐trivial task. This study compares five different methods to integrate a reference electrode into an alkaline zero‐gap CO_2_ electrolysis cell. Sources of error and measures to circumvent them are investigated and finite‐element simulation is used to gain a better understanding of observed effects. Placing a reference electrode into the inactive area of the cell is found to be a reliable method, as long as the placement of electrodes is sufficiently controlled. Sandwiching a wire quasi‐reference electrode between two membranes is especially useful for electrochemical impedance spectroscopy; however, it can affect the overall cell performance. Contacting the catalyst layer from the backside with a salt‐bridge is promising for localized measurements if sufficient reproducibility can be ensured.

## Graphical abstract

Integrated reference electrodes can offer valuable insights into processes during zero‐gap CO_2_ electrolysis, required for further improvement. Different methods of integration, however, present unique benefits and limitations. This work compares five different methods based on experimental results and numerical simulation, highlighting practical challenges, and providing a guide for choosing a suitable setup.

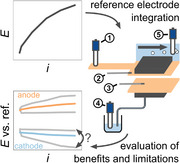

## 1 Introduction

CO_2_ electrolysis is a promising technology for producing fossil‐free feedstock chemicals from renewable electrical energy, carbon dioxide and water. The field has progressed significantly within the last years, with electrolyzers pushing for energy efficiencies beyond 40 % for carbon monoxide as target product.^[^ ^1^, ^2^, ^3^ ^]^ While there is an ongoing debate about the ideal cell design, zero‐gap electrolyzers using an anion‐exchange membrane and gas‐fed cathode can be considered state‐of‐the‐art, as they provide high product selectivity, low ohmic resistances and high reaction rates.^[^ ^4^ ^]^

Several recent reviews conclude, that to make further progress in terms of cell performance, advanced operando techniques are required to gain sufficient insight into the processes taking place in the cell.^[^ ^5^, ^6^, ^7^ ^]^ One way to gain more insight into full cell experiments is to integrate a reference electrode. This allows to deconvolute each electrode's contribution to the full cell voltage.

Integrating a reference electrode, even though common for CO_2_ electrolyzers using a catholyte, is particularly challenging for the zero‐gap setup. Firstly, the setup does not provide a lot of space for placement of an additional electrode. Secondly, the integrated reference electrode should not affect cell operation and has to be stable and reliable for the duration of the experiment. Several groups presented integrated reference electrodes in a zero‐gap CO_2_ electrolyzer in recent years.^[^ ^8^, ^9^, ^10^, ^11^ ^]^ However, the focus is generally on the study and not the reference electrode setup itself. Studies comparing different reference electrode setups, highlighting challenges and assessing the quality of results are yet not available. Choosing a setup can thus be challenging for researchers new to the field.

**Figure 1:**
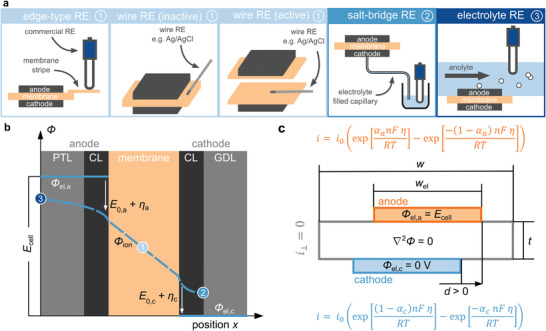
Scope of this work: a) Overview of the five investigated setups for integrating a reference electrode (RE); b) 1D potential distribution through a CO_2_ electrolysis cell and indicated contact points of each setup under idealized assumptions; c) Scheme for 2D finite element simulation of the potential distribution in the membrane of a CO_2_ electrolysis cell.

Generally, a lot of knowledge on the integration of reference electrodes exists in fields outside of CO_2_ electrolysis. The topic has been discussed for batteries,^[^ ^12^ ^]^ fuel cells^[^ ^13^, ^14^, ^15^, ^16^, ^17^ ^]^ and various types of water electrolyzers.^[^ ^18^, ^19^, ^20^, ^21^, ^22^, ^23^, ^24^, ^25^ ^]^ Many similarities exist between the presented setups, as they usually belong to one of five setup types, as shown in **Figure** **1**a: edge‐type setup, inactive wire setup, active wire setup, salt‐bridge setup and electrolyte setup. The key to integrating a reference electrode despite the geometrical constraints is to either contact the ionic phase of the cell to the outside and use a commercial reference electrode or to miniaturize the reference electrode itself by using a wire reference electrode.

The one‐dimensional potential distribution throughout the electrolysis cell (as illustrated in Figure 1b) serves as a helpful simplification for comprehending and interpreting measurement outcomes across various setups.^[^ ^19^, ^20^, ^26^ ^]^ Due to the comparably high electrical conductivity of the porous transport layers and catalyst layers, the electrical potential *Φ*_el_ can be assumed to be constant within these layers. The reference electrode senses the ionic potential *Φ*_ion_. The exact contact point within the system depends on the utilized setup. Under idealized assumptions, the edge‐type setup and both inactive and active wire setups provide access to the ionic potential in the middle of the membrane (1). However, the idealized assumptions prove to be false for many practical cases, as will be discussed below. A salt‐bridge from the cathode side allows contacting the ionic potential at the backside of the cathode catalyst layer (2). A reference electrode placed in the anolyte provides access to the backside of the anode porous transport layer (3). Thus, all five setups are in theory able to provide information about anode and cathode contributions to the overall cell performance.

When confronted with a data set from a setup using an integrated reference electrode, it can be challenging to determine, if the data is error‐free and correctly represents the average anode and cathode potentials. A very good understanding of the system can help to identify erroneous data, but the required level of understanding can often only be obtained by using a working reference electrode setup in the first place. The most practical approaches to determining the quality of a setup are thus testing its reproducibility^[^ ^20^ ^]^ and using qualitative numerical simulation to understand possible sources of error and intentionally inducing these error sources in the real setup.^[^ ^14^, ^27^, ^28^, ^29^, ^30^ ^]^ To provide an understanding of failure mechanisms, we provide numerical finite element simulation to observed errors in measurements (Figure 1c). Similar to other simulations in the literature, Laplace's law is used as governing equation and electrode kinetics are described by Butler‐Volmer‐Equation.^[^ ^14^, ^27^, ^28^ ^]^ A central parameter investigated is the electrode misalignment factor *d*/*t*, describing the ratio between electrode displacement *d* and membrane thickness *t*, as illustrated in Figure 1c. An extensive description of all simulations is presented in the supplementary information.

The required accuracy for data measured with an integrated reference electrode has to be determined in the context of the electrochemical cell, which is investigated. We estimate 50 mV to be a reasonable accuracy for zero‐gap CO_2_ electrolyzers. Figure S1 (Supporting Information) shows the reproducibility of experiments in a two‐electrode setup, which falls into this range. This sets a baseline for the accuracy we can expect when adding an additional electrode. In experiments comparing different catalyst layers, a setup with this level of accuracy should be sufficient to observe significant differences in activity. Additionally, the potential of the reference electrode should not change more than 50 mV over the duration of a measurement, which has to be considered for lifetime experiments. For other technologies, it may be necessary to reevaluate this value.

The aim of this study is to provide a guide to the integration of reference electrodes and help the reader to choose a suitable setup. Common issues of each setup are highlighted and ways to circumvent them are discussed. Even though the work is primarily focused on the implementation in CO_2_ electrolyzers, we believe that many aspects can be transferred to related technologies.

## 2 Results and Discussion

### 2.1 Edge‐Type Reference Electrode

In the so‐called edge‐type setup, the reference electrode is placed outside of the active cell area with an ionic connection to the membrane. This connection can either be facilitated by (i) integration of the reference electrode in the cell fixture and putting it in direct contact with the membrane^[^ ^10^, ^31^ ^]^ or (ii) by using an additional membrane strip as extension to connect the reference electrode outside of the cell fixture.^[^ ^14^, ^24^, ^25^ ^]^ In this study, the membrane strip option (ii) was employed, as illustrated in Figure 1a. To investigate the effect of electrode misalignment, two reference electrodes (RE_1_ and RE_2_) were integrated at opposing sides of the cell. An exploded view of the setup, including all components, can be found in Figure S2 (Supporting Information).

**Figure 2:**
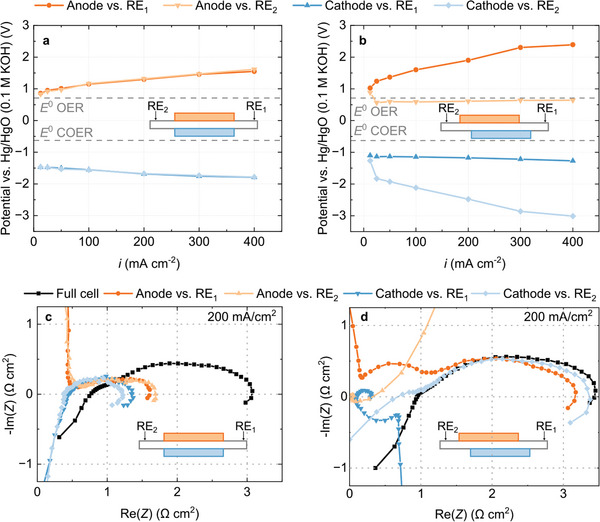
Experimental results obtained from the edge‐type setup. a) Anode and cathode potentials measured with electrodes positioned on top of each other and b) with electrodes misaligned by 1 mm. c) Full cell and half‐cell impedance spectra taken at 200 mA cm^−2^ with aligned electrodes and d) with misaligned electrodes.

**Figure 3:**
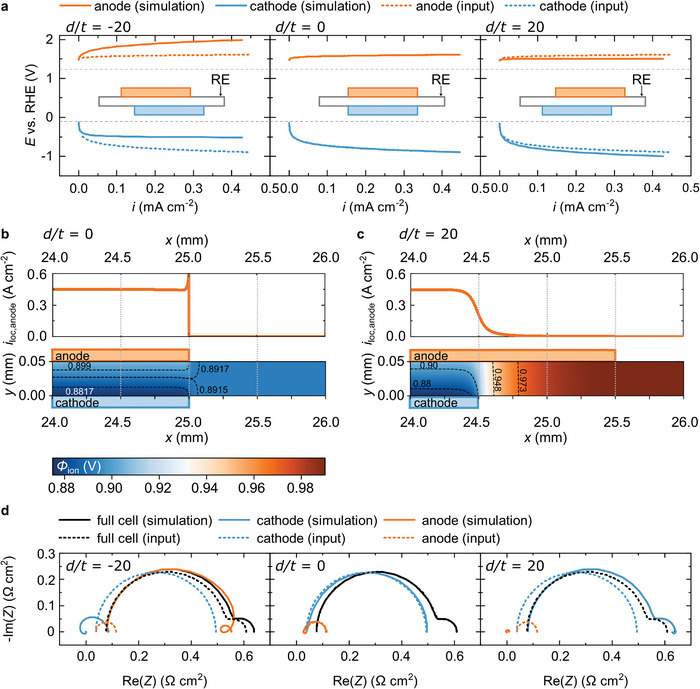
Simulated effect of electrode misalignment on measured electrode potentials and impedance spectra. a) Simulated electrode potentials for different degrees of electrode misalignment; b) local current at the anode (i_loc,anode_) and potential distribution at the edge of the catalyst layer with aligned electrodes and c) with misaligned electrodes at 2.5 V cell voltage. d) Simulated impedance spectra for different degrees of electrode misalignment at 2.4 V cell voltage.

**Figure** **2**a shows measured electrode potentials versus both reference electrodes when anode and cathode are positioned on top of each other. Measurements versus both reference electrodes yield matching data sets. The differences in both signals are below 50 mV, indicating that the alignment can be controlled sufficiently. However, if the electrodes are misplaced by 1 mm (corresponding to a misalignment factor *d/t* of 20 at 50 µm membrane thickness), different potentials are measured versus RE_1_ and RE_2_ (Figure 2b). The potentials are more positive when measured against RE_1_ than RE_2_. Additionally, the change in cathode potential versus RE_1_ is small, whereas the change in anode potential is small versus RE_2_. The effect that misplaced electrodes have on a measurement in this type of setup is a well‐known phenomenon.^[^ ^14^, ^27^ ^]^ Both data sets are flawed because one electrode is closer to the reference electrode than the other. This leads to contradicting results as RE_1_ overestimates the contribution of the anode and RE_2_ overestimates the contribution of the cathode to the overall cell potential. A simulation based explanation of the phenomenon is provided below (**Figure** **3**).

We observe a similar behavior in electrochemical impedance spectra. If the electrodes are aligned (Figure 2c), measurements against RE_1_ and RE_2_ result in matching data sets. The measurement with aligned electrodes reveals that under these operating conditions, anode and cathode contribute to a similar degree to the overall cell impedance. However, if the electrodes are misaligned, the half‐cell spectra obtained from both reference electrodes differ strongly (Figure 2d). Measured versus RE_1_, the anode appears to make up for a large portion of the full impedance. Measured against RE_2_, however, the cathode appears to be responsible for a large portion of the impedance.

At frequencies beyond 20 kHz, large artifacts distort the half‐cell spectra. This distortion occurs for all setups but the active wire reference electrode (see Figure S3, Supporting Information). Bühre et al. reported similar artifacts in the same frequency range for polymer electrolyte membrane water electrolyzers.^[^ ^20^ ^]^ The reason for this artifact is unknown. We suggest that the artifacts are related to the way the reference electrodes are integrated and not to external circuitry, because they do not appear in full cell spectra. However, further research should be focused on the reason behind the artifacts and their impact on half‐cell spectra, before these measurements are utilized to learn more about the electrolysis cells. The artifacts can heavily obscure especially the high‐frequency resistance.

Several articles have discussed the effect of misaligned electrodes on experimental data with a reference electrode placed in the inactive area for fuel cells.^[^ ^14^, ^27^, ^28^ ^]^ While our simulation confirms the already reported findings, we would like to emphasize some specific characteristics and their implications on measurements and attempts to correct for measurement error due to misalignment.

Figure 3a compares simulated measurements of electrode potentials for misalignment factors *d/t* equal to −20, 0 and 20 (corresponding to ‐1 mm, 0 mm and 1 mm electrode misalignment). The dotted lines indicate the expected potentials based on the input parameters of the simulation (electrode kinetics and membrane resistance). Section S1 in the supporting information provides a detailed description of the simulation. The simulation confirms that a protruding cathode (*d/t* = −20), meaning that the cathode extends further towards the reference electrode than the anode, leads to a shift toward more positive values. If the anode is protruding (*d/t* = 20), the potentials are more negative. The measured data is only correct, if there is no misalignment. Additional simulations testing smaller displacement factors (Figure S4, Supporting Information) show that the error can be reasonably small when the misalignment is smaller than the membrane thickness. Thus, in an experimental setup the alignment does not have to be perfect, but just sufficiently well controlled.

Figure 3b,c help to rationalize why different electrode potentials are measured, depending on the electrode alignment. Both consider the potential distribution within the membrane and the local current drawn from the anode within the transition area between electrode and inactive area of the cell. Figure 3b describes a cell with aligned electrodes, which has been discussed extensively in the literature.^[^ ^14^, ^27^ ^]^ Generally, the potential becomes virtually constant at a distance more than three times the membrane thickness (*d* > 3*t*) from the active area.^[^ ^27^ ^]^ It is equal to the potential in the active area halfway through the membrane. This way, the contribution of ohmic losses in the membrane distributes equally to the measured anode and cathode potential, resulting in a well‐defined reference potential. At the anode, current is drawn at a constant level up to the edge of the electrode.

In contrast, if the electrodes are misaligned (Figure 3c) the potential sensed by the reference electrode in the inactive area is much higher than any potential, which can be found in the active area of the cell as shown by He et al. previously.^[^ ^14^ ^]^ The reason for this is a small local current, which can be drawn from the anode in places where the anode is protruding and the cathode is discontinued. This local current is lower than the current in the active area, because it has to overcome a larger distance in the lateral direction of the membrane. Because of the reduced current, the local overpotential of the anode is smaller in the inactive area than in the active area. Thus, the protruding part of the anode biases the membrane potential toward a more positive potential. This leads to a shift of the reference potential to more positive values, which makes both the anode and cathode potential seem more negative in comparison.

Notably, the absolute voltage by which the potentials are shifted towards positive or negative values is not identical in Figure 3a, even though the electrodes are displaced by 1 mm in both the left and right graph. The shift of the potential is not symmetrical, because the kinetics of the protruding electrode determines how much the reference potential is biased and the anode and cathode have different kinetics. Due to the higher tafel slope at the cathode, a protruding cathode affects the potentials stronger than a protruding anode in this case. As a result it is not possible to correct the data set obtained with two reference electrodes placed at opposing sides of a misaligned cell by taking the average of both data sets, when both electrodes show significant differences in kinetics.

Some simulations in literature assume primary current distributions.^[^ ^27^, ^28^ ^]^ This means electrode kinetics are neglected and a uniform ionic potential at the electrode is assumed. Under this assumption, the simulated potential in the inactive parts of the cell is always within the bounds of the potentials in the active area. Additionally, the shift in measured electrode potentials appears to be a purely ohmic effect. Based on this understanding, two methods for correcting measured data were suggested: (i) intentionally misaligning electrodes so that the potential in the inactive are is equal to the potential found at one of the electrodes^[^ ^8^, ^32^ ^]^ and (ii) using the half‐cell high frequency resistance for *iR*‐compensation.^[^ ^13^, ^20^, ^24^ ^]^ Both methods seem valid, if a primary current distribution is assumed. However, we argue that the electrode kinetics play a significant role and a secondary current has to be assumed. Consequently, intentional misalignment does not work. If electrodes are misaligned in an attempt to shift all voltage losses caused by the membrane resistance to the signal of one electrode, one inevitably causes additional error due to the kinetics of the protruding electrode material.

*iR*‐compensation can help to a limited degree, because the shift in measured potentials does have an ohmic part. However, when correcting the data from Figure 2a by the high frequency resistance, the signals measured versus RE_1_ and RE_2_ are still significantly different (see Figure S5, Supporting Information). Generally, *iR*‐compensation is more effective, if the contribution of ohmic losses are large compared to kinetic losses, i.e., if the membrane resistance is high or electrode kinetics are fast (see Figure S6, Supporting Information). Using *iR*‐compensation can have a positive effect on data quality, especially in applications with faster kinetics, like polymer electrolyte membrane water electrolysis. However, it is not a rigorous method for correction and clearly insufficient in the case of the CO_2_ electrolysis cells investigated here.

Figure 3d shows the effect of misalignment on impedance spectra. For an in‐depth discussion we refer to Cimenti et al.^[^ ^29^, ^30^ ^]^ and Ender et al.^[^ ^12^ ^]^ Misalignment causes a large portion of the cell impedance to apparently shift from anode to cathode. The fundamental reason behind the errors in impedance spectra is a frequency dependent change in the potential distribution within the membrane.^[^ ^30^ ^]^ Both groups found, that a mismatch between the electrodes, either by geometrical misalignment or due to different kinetics, causes distortions. Geometrical misalignment causes the semicircle of the electrode, which extends closer to the reference electrode, to shrink and its high frequency intercept to shift to smaller values.^[^ ^12^, ^30^ ^]^ This can also be observed in Figure 3d for *d/t* = 20, where the anode semicircle becomes very small and lies close to zero. However, even with perfect alignment, the measured spectra only get close to the expected spectra based on the input parameters. Differences in the electrode kinetics cause inductive artifacts at high frequencies for one electrode and at low frequencies for the other. Thus, the spectra are slightly deformed from the semicircular shape.^[^ ^12^, ^29^ ^]^ The combination of different electrode kinetics and misalignment causes cross‐contamination, which can be most easily seen for *d/t*  =  20, where an additional semicircle appears in the cathode spectrum.

To summarize, the simulation suggests, that the quality of impedance spectra is high, but not perfect, if both electrodes are properly aligned. However, it remains unclear, how well the simulation translates to the experiments and how significant the impact of the simulated effects is in the real application. We propose that impedance spectra taken with aligned electrodes can be utilized for qualitative statements. However, relying on impedance spectra for quantitative results requires further research on the magnitude of the remaining errors due to different electrode kinetics or the artifacts observed at high frequencies.

Lastly, we would like to give some practical remarks for conducting experiments with this setup. Humidifying the membrane strip ensures a sufficient ionic contact. Dry‐out of the strip usually becomes immediately visible, as the reference potential starts to float and the measured electrode potentials shift in parallel (see Figure S7a, Supporting Information). When properly humidified, the membrane strip (Aemion+ membrane AF2‐HNN8‐50‐X, 50 µm thickness) has a resistance of roughly 50 kΩ (Figure S7b, Supporting Information).

The membrane strip has to be completely isolated from all electrically conductive components of the cell. Only very little current flows through it toward the reference electrode, thus any other Galvani potential along the connection to the reference electrode can disturb the potential sensed by the reference electrode heavily, which leads to a strong shift in the measured electrode potentials. This can be demonstrated experimentally, by bringing the membrane strip in direct contact with either anode or cathode flow field (Figure S7c,d, Supporting Information). The effect can also be demonstrated in the simulation by inserting an additional electrode with slow kinetics along the membrane strip (Figure S8, Supporting Information). We present a more detailed discussion of the effect below in the context of the salt‐bridge reference electrode setup.

To generate a breakdown of voltage losses (i.e., electrode overpotentials) based on data obtained from a reference electrode setup, precise knowledge of the standard potentials *E* ^0^ of each electrode is required. In practice, this can be challenging. It is possible to estimate *E* ^0^ based on literature values and the bulk pH of the anolyte. This is the method used to determine *E* ^0^ in this work, as indicated in Figure 2a,b (Section S2, Supporting Information). However, the local environment of the electrode is largely unknown and the local pH may differ significantly.^[^ ^33^ ^]^ Ideally, *E* ^0^ would be determined in‐situ. Xu et al.^[^ ^24^ ^]^ suggested such a method for anion‐exchange membrane water electrolyzers. By forming hydrogen at the cathode and immediately measuring the open circuit voltage, they effectively build an in situ RHE, which can be used for calibration. Due to the slow kinetics of the silver‐based electrodes in this work, the concept could not be transferred to the CO_2_ electrolyzer. The electrodes reacted sluggishly, leading to a strongly drifting signal, which could not be used to determine *E* ^0^. Without this type of calibration method, the uncertainty in *E*° can add additional error to a voltage breakdown.

As shown before, good electrode alignment is crucial to obtain useable data with this setup. Practically, we were only able to ensure the degree of alignment when using gas diffusion electrodes. During the fabrication of catalyst coated membranes, solvents caused the membrane to swell and the electrodes were regularly displaced by up to 1 mm (Figure S9, Supporting Information). If the membrane material does not require an ion‐exchange and can thus be assembled in dry state, it may be possible to achieve the required geometrical control, for example by using laser‐ablation to shape both catalyst layers to identical size.^[^ ^13^, ^34^ ^]^ However, with the materials used in this work, the utilization of the edge‐type setup is restricted to gas diffusion electrodes.

### 2.2 Wire Reference Electrode (Inactive)

As illustrated in Figure 1a, the wire reference electrode configuration uses a miniaturized quasi‐reference electrode, which is placed inside the fixture in direct contact with the membrane. “Inactive” describes the placement of the wire electrode outside of the active cell area. In this work, a silver/silver chloride wire electrode is utilized as quasi‐reference electrode (see Figure S10, Supporting Information). The wire reference electrode concept was adapted from Hansen et al.,^[^ ^8^ ^]^ using a silver chloride electrode instead of a silver bromide electrode. The electrode was fabricated by coating a silver wire with a silver chloride layer followed by a protective overcoating, consisting of a cation exchange polymer. In ex‐situ experiments, the electrode shows the behavior expected of a regular silver/silver chloride reference electrode (see Figure S11, Supporting Information) and achieves the required accuracy below 50 mV. However, when placing the wire reference electrode in the cell, the local concentration of Cl^−^ anions and thus the exact reference potential is unknown. Therefore, the potential of each wire reference electrode was measured against a commercial mercury oxide electrode placed in the anolyte before each experiment. This way, experimental data from this setup can be reported against a mercury oxide electrode and be compared to data obtained from other setups.

**Figure 4:**
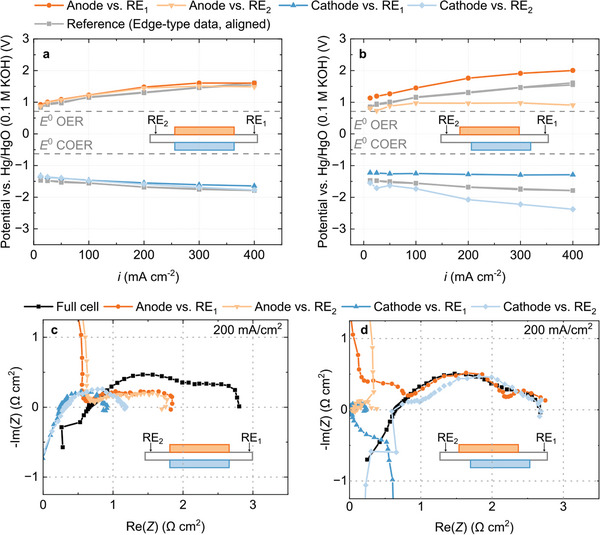
Experimental results obtained from the wire reference electrode (inactive) setup. a) Anode and cathode potentials measured with electrodes positioned on top of each other and b) with electrodes misaligned by 1 mm. c) Full cell and half‐cell impedance spectra taken at 200 mA cm^−2^ with aligned electrodes and d) with misaligned electrodes.

**Figure** **4**a,b show measured electrode potentials obtained from a cell with two integrated wire reference electrodes with aligned and misaligned electrodes. The results are similar to the results obtained in the edge‐type configuration. Misaligned electrodes lead to a shift in electrode potentials and only if the electrodes are precisely aligned, the data sets measured against both reference electrodes line up. Additionally, the data obtained from the inactive wire reference electrode setup with aligned electrodes is comparable to the results from the edge‐type setup.

Figure 4c,d show the electrochemical impedance spectra against RE_1_ and RE_2_ with aligned and misaligned electrodes. Similar to the results from the edge‐type setup, misaligning the electrodes leads to an inflation of one electrode's half‐cell spectrum and a deflation of the other electrode's spectrum. Only if the electrodes are aligned, the two measurements give similar results.

The inactive wire reference electrode setup is functionally very similar to the edge‐type setup. Consequently, all learnings from Figure 3 discussed in the previous section are transferrable to this setup. In summary, the most important points are that the measured data under direct current conditions is only correct, if the electrodes align properly. Intentional misalignment and *iR*‐compensation are not suitable to correct the error of misaligned electrodes. With the materials used in this work, the setup is restricted to using gas diffusion electrodes. Electrochemical impedance spectra contain artifacts even with aligned electrodes. The magnitude of the error is not well understood for practical experiments, thus the data should only be used for qualitative statements.

The potentials measured in a cell with aligned electrodes line up less good in this setup compared to the edge‐type setup. The reason for this is the instability of the wire potential, once it is placed in the cell. To examine this, the stability of the wire potential was investigated after each fabrication step (see Figure S12, Supporting Information). The potential was most stable once the protective polymer layer was cured in the oven. The change in the reference potential during the testing was then found to be about 50 mV. In some tests, a change of up to 200 mV was observed in the reference potential. Thus, further improvement of the wire reference electrode is required to increase its stability and ensure the required accuracy of 50 mV.

### 2.3 Wire Reference Electrode (Active)

The previously discussed setups are highly sensitive to the misalignment of the electrodes. Placing the reference electrode within the active area of the cell can help to avoid this challenge.^[^ ^22^ ^]^ In this work, this is achieved by sandwiching the wire reference electrode between two membranes, which have half the thickness (25 µm) of the membrane used in the remaining setups (50 µm), as shown in Figure 1a. This makes it the only reference electrode that interferes with the current of the electrolysis cell. An exploded view of this setup can be found in Figure S11 (Supporting Information). The simulation confirms that electrode misalignment has only a minor impact on the measured electrode potentials and impedance spectra in this setup. This can be attributed to the reduction in the effective cell area, which results in smaller currents and manifests as a negligible effect in the measurement results (see Figure S14, Supporting Information).

**Figure 5:**
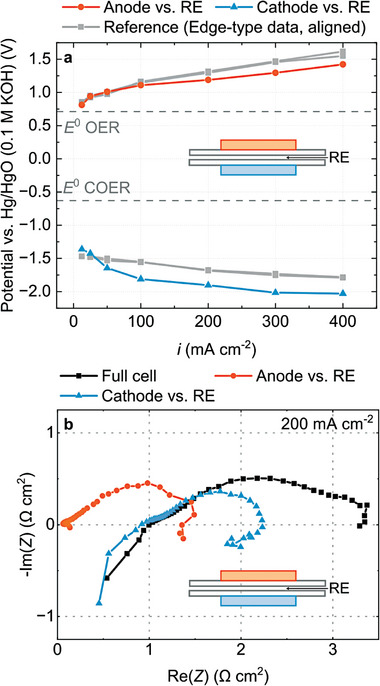
Experimental results obtained from the wire reference electrode (active) setup. a) Anode and cathode potentials and b) full cell and half‐cell impedance spectra taken at 200 mA cm^−2^.

**Figure** **5**a shows electrode potentials measured with the “active” wire reference electrode. At low current densities, the results are comparable with the results obtained from the edge‐type reference electrode. At higher current densities above 100 mA cm^−2^, however, they differ by about 200 mV. The signal obtained from the wire reference electrode in the active cell area is noisier than the signal from the inactive one (see Figure S15, Supporting Information). This raises the concern that the transport of species between anode and cathode affects the potential of the quasi‐reference electrode placed between them. Hartig‐Weiß et al.^[^ ^22^ ^]^ used a platinum wire as a reference electrode in a polymer electrolyte membrane water electrolyzer. They found, that the potential of the reference is dependent on the flux of O_2_ and H_2_ through the membrane and thus on the operating point. They concluded that measurements of electrode potentials were only valid at low current densities.^[^ ^22^ ^]^ In the herein presented system a complex mixture of species, including CO_2_, H_2_, CO, O_2_, OH^−^, CO_3_^2−^, HCO_3_^−^, H_2_O and K^+^ can be present in the membrane and interfere with the potential of the quasi‐reference electrode. Furthermore, it is possible that transport processes between anode and cathode remove ions from the local environment of the reference electrode (Ag^+^ or Cl^−^), thus affecting its potential. The unstable data obtained in this setup indicates that the reference potential is somehow destabilized. We suggest further research into the causes of this destabilization or alternative electrodes before utilizing the “active” wire reference electrode setup for measuring electrode potentials over a large range of current densities.

**Figure 6:**
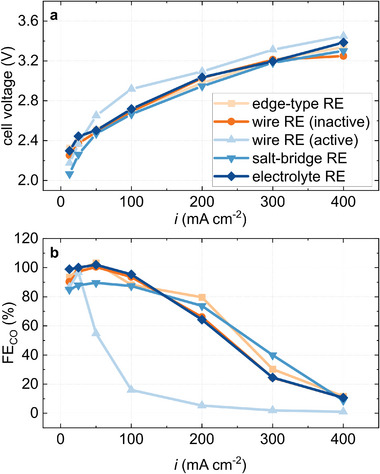
Full cell performance with integrated reference electrodes. a) Cell voltage and b) faradaic efficiency towards CO at each current step.

Moreover, the “active” wire reference electrode setup affects the overall cell performance negatively. Compared to the other setups, the cell voltage increased and the selectivity towards carbon monoxide starts decreasing already at current densities above 25 mA cm^−2^ (see **Figure** **6**). One likely reason for the negative effect on the cell voltage might come from the isolation of the wire reference electrode. To ensure that the wire electrode is only in contact with the active area of the cell, a piece of Kapton‐tape surrounds the electrode and is inserted by 1 mm into the active area (see Figure S13, Supporting Information). By further developing the wire electrode (e.g., using a thin PTFE wrapping for this isolation), the impact of the reference electrode on the cell performance can probably be decreased.

For electrochemical impedance spectroscopy the required stability of the reference potential is lower, because of the shorter duration of the experiment.^[^ ^22^ ^]^ A quasi‐reference with an uncertain potential can be utilized, as long as it is stable during the entire experiment. The impedance spectra obtained from this setup (see Figure 5b) show comparable semicircles for anode and cathode half‐cell spectra, which was also observed in previous setups. Notably, this setup does not show an artifact at high frequencies for half‐cell spectra (see Figure S3, Supporting Information). This facilitates the determination of high frequency resistances and makes the setup more suitable for electrochemical impedance spectroscopy than other setups.

In summary, this setup requires further development if used to determine electrode potentials. The choice of wire electrode may not be ideal. The stability of the reference potential should be further investigated and the wire should be inserted in a way that reduces its influence on the cell performance. Despite these shortcomings, the “active” wire setup shows very promising properties for electrochemical impedance spectroscopy, because it is not influenced by electrode misalignment and does not show artifacts at high frequencies.

### 2.4 Salt‐Bridge Reference Electrode

The salt‐bridge reference electrode setup employs a capillary salt bridge to establish ionic contact with the backside of the cathode (see Figure 1a). By doing so, the contact does not interfere with the transport processes between the anode and cathode and it is positioned within the active area of the cell. Consequently, the setup is insensitive to electrode misalignment (see Figure S14, Supporting Information). Brightman et al.^[^ ^18^ ^]^ demonstrated that it is possible to measure even local potentials at various locations of a fuel cell cathode, by using this configuration. This allows for the investigation of the electrode potential distribution along the cell area, which is a capability unique to this setup. So far this configuration has been utilized in fuel cells and electrolysis cells,^[^ ^15^, ^18^, ^19^, ^21^, ^35^ ^]^ but not in CO_2_ electrolysis. In the salt‐bridge reference electrode setup used in this work, a capillary filled with 1 м KOH serves as salt bridge between the cathode and an external reference electrode (see Figure S16, Supporting Information).

The main challenge of this setup is to establish sufficient ionic contact between the capillary and the catalyst layer through the gas diffusion layer. Methods to achieve this are (i) punching a hole in gas diffusion layer and catalyst layer and bringing the capillary in direct contact with the membrane;^[^ ^21^ ^]^ (ii) punching a hole through the gas diffusion layer and bringing the capillary in contact with the catalyst layer or (iii) impregnating gas diffusion layer with an ionomer solution to establish the contact.^[^ ^15^, ^19^ ^]^ Piela et al.^[^ ^35^ ^]^ discussed all three options for a fuel cell. Punching a hole through gas diffusion layer and catalyst layer (i) interrupts the electrode at the contact point, causing the anode to extend in a position, where no cathode is present. This is a special case of the error caused by misalignment in the edge‐type setup. Thus, it has a similar effect on the measured potentials, as visualized in Figure S17 (Supporting Information). When the hole is punched only through the gas diffusion layer (ii), both the reactant transport and the electrical contact at the sensing point are affected negatively. This reduces the local activity of the catalyst and affects the measurement result in a similar way as case (i).^[^ ^35^ ^]^ Therefore, the impregnation of the gas diffusion layer with an ion‐conductive binder (iii) remains as the only viable option.

**Figure 7:**
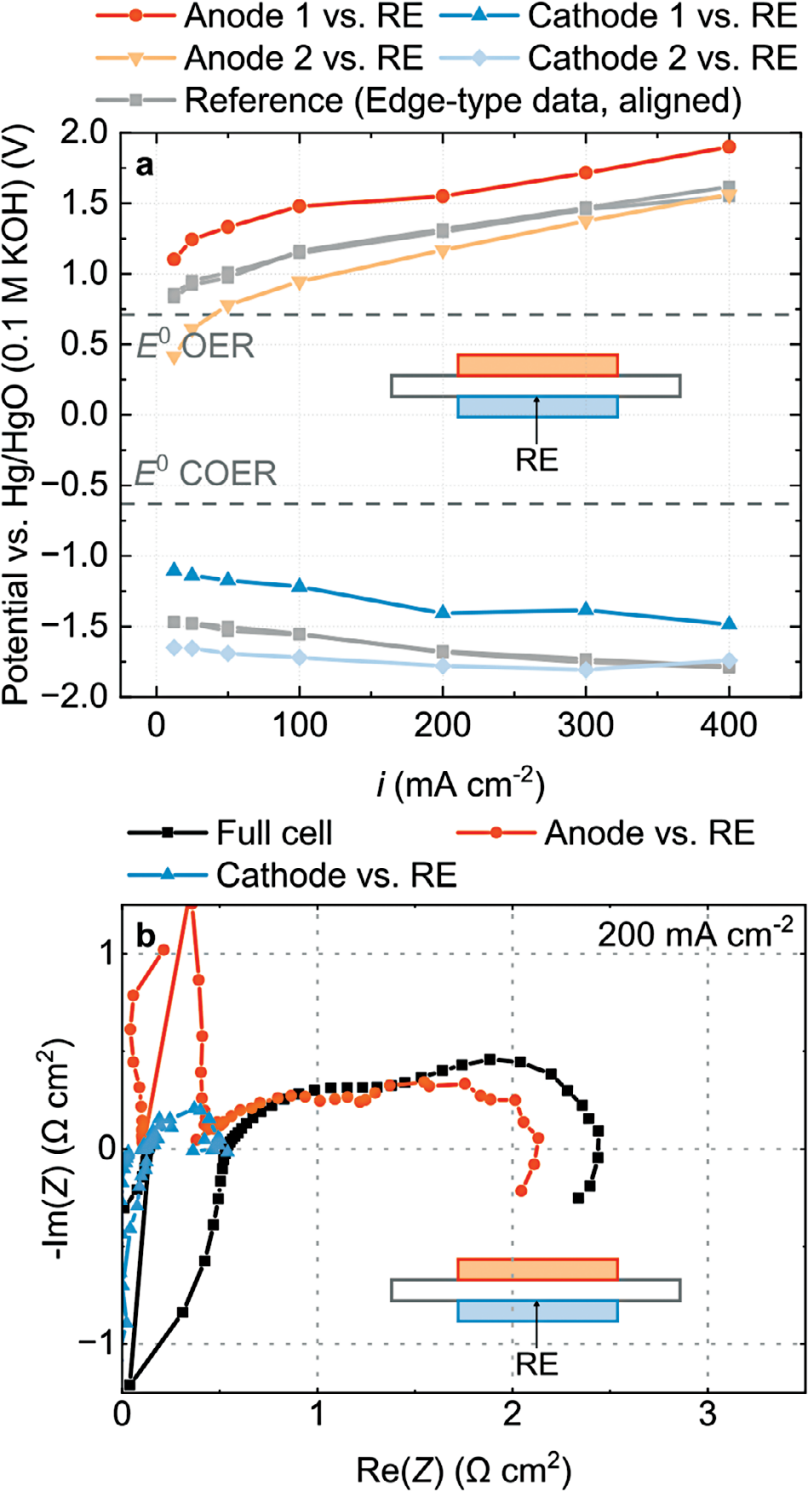
Experimental results obtained from the salt‐bridge reference electrode setup. a) Anode and cathode potentials from two tests using identical cells and b) full cell and half‐cell impedance spectra taken at 200 mA cm^−2^.

**Figure** **7**a shows that even when identical cells were used, we observed significant deviations between two test runs. Compared to the data obtained in the edge‐type setup, the electrode potentials could be both shifted towards positive or negative values. Figure 7b shows the impedance spectra obtained from this setup. A large share of the impedance seems to originate from the anode, which contradicts the results from all previous setups. We suspect the reason for the unreliable measurements to be the impregnation of the gas diffusion layer, as will be discussed in the following.

**Figure 8:**
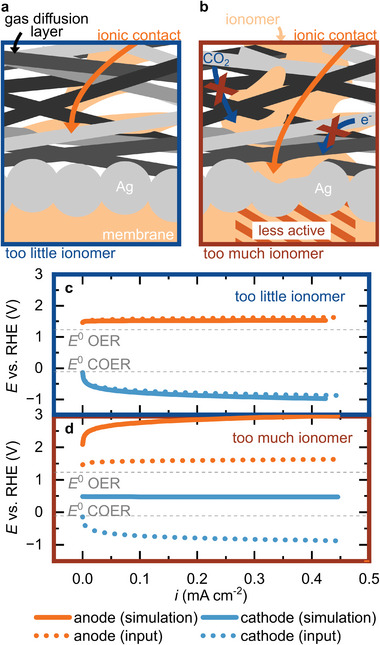
Error mechanisms in the salt‐bridge reference electrode setup. a) Sketch of too little impregnation and b) too much impregnation. c) Simulated electrode potentials if the ionic contact is insufficient due to too little impregnation and side reactions are sensed and d) if the catalyst is locally less active due to too much impregnation.

The gas diffusion layers best suited for CO_2_ electrolysis typically use a microporous layer,^[^ ^36^ ^]^ which also has to be impregnated. However, the microporous layer complicates impregnation. To establish an ionic contact in the first place, a contraption, which uses a vacuum to pull the ionomer solution through the gas diffusion layer, was used (Figure S18, Supporting Information). This crude fabrication process does not ensure that the contact is of equal quality for every sample. It is possible to either impregnate the sample too much (**Figure** **8**a) or too little (Figure 8b).

Too little ionomer causes insufficient ionic contact to the catalyst layer. Thus, any electrochemical process in proximity to the ionic contact can interfere with the potential sensed by the reference electrode. Instead of the electrode potentials of anode and cathode, the potential of this side reaction is sensed. Becker et al.^[^ ^37^ ^]^ used this effect to measure corrosion potentials in polymer electrolyte membrane water electrolyzers. As a consequence, the recorded electrode potentials appear to be shifted, possibly even beyond their standard electrode potential, and the anode shows a constant potential independent of the working point (Figure 8c). The simulation and further considerations are presented in Figure S19 and Section S3 (Supporting Information).

Too much ionomer impregnation has similar consequences to punching a hole through the gas diffusion layer as discussed above. The excess ionomer may block reactants and an ionomer film on the gas diffusion electrode may reduce the electrical contact. Consequently, the catalyst layer is less active at the contact point. A simulation of the error in measured electrode potentials is presented in Figure 8d.

In summary, the salt‐bridge setup is insensitive to electrode misalignment and allows local potential measurements. Although it has been successfully employed in fuel cells and water electrolysis, its implementation in a zero‐gap CO_2_ electrolyzer is not straightforward, due to different employed materials and operating conditions. In particular it is challenging to facilitate a sufficiently good ionic contact through the gas diffusion layer, which does not affect the result of a measurement, because too much or little impregnation can lead to erroneous results. This results in low reproducibility with the materials used in this work. However, there is potential for optimization of the impregnation process (e.g., amount and dilution of the ionomer solution) and a different choice of gas diffusion layer or of ionomer for impregnation may improve the reproducibility.

### 2.5 Electrolyte Reference Electrode

The electrolyte reference electrode setup is the simplest way to integrate a reference electrode.^[^ ^9^, ^11^ ^]^ In this setup, a commercial reference electrode is placed in the anolyte stream (see Figure 1a; Figure S20, Supporting Information). The electrode has to be placed at the inlet, to prevent gas bubbles from disconnecting the reference electrode. To establish sufficient ionic contact, the anolyte has to be sufficiently conductive. Similar to Murakami et al.^[^ ^9^ ^]^ we found that a minimum electrolyte concentration of 0.1 м KOH was required to obtain a stable signal. Simulations indicate that electrode misalignment does not play a role in this setup (see Figure S14, Supporting Information).

**Figure 9:**
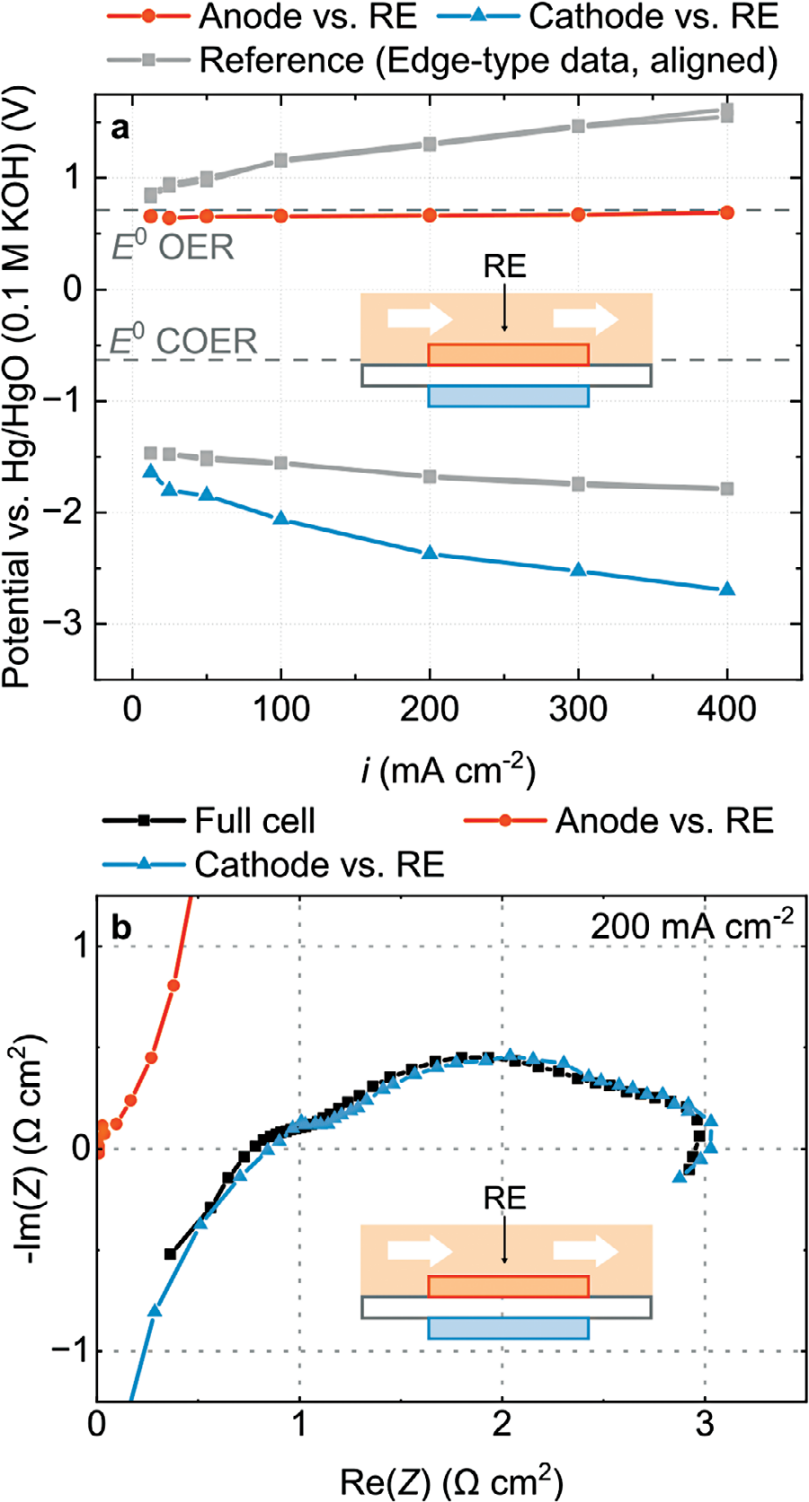
Experimental results obtained from the electrolyte reference electrode setup. a) Anode and cathode potentials and b) full cell and half‐cell impedance spectra taken at 200 mA cm^−2^.

However, **Figure** **9**a shows electrode potentials that strongly differ from that of other reference setups. The anode potential does not change with increasing current. All change in the cell voltage appears to be caused by the cathode. Also the impedance spectra in Figure 9b indicate that the cathode causes all of the cell impedance and the anode does not contribute any impedance. From the other reference electrode setups, it is clear, that this observation is not correct for the presented system.

As indicated in Figure 1b, this setup contacts the cell at the backside of the porous transport layer. However, the ionic potential is not constant throughout the depth of the electrode. A higher faradaic current is drawn from the region close to the membrane, than from the region at the backside of the electrode because the ionic resistance of the electrode is higher than its electric resistance. The higher faradaic current leads to a higher overpotential close to the membrane than at the backside of the electrode (Figure 1b).^[^ ^26^ ^]^ Thus, it is not possible to separate the entire overpotential of the anode by using a contact point at the backside of the porous transport layer. A large portion of the anode's overpotential is measured between reference electrode and cathode in this case. This can also be seen in the experimental data, where the anode potential hardly changes and is close to its standard electrode potential. All changes in the cell voltage appear between cathode and reference electrode.

This observation is also confirmed in other publications, where the voltage measured between anode and a reference electrode in the anolyte also tends to be constant at various operating points.^[^ ^9^, ^11^, ^26^ ^]^ Therefore, the contact point of the reference electrode is inadequate to correctly separate the anode potential from the cathode potential, which is the central drawback of this setup. While it can be used in combination with other setups to gain more information about the voltage drop across the depth of each electrode,^[^ ^26^ ^]^ we do not recommend this setup as a standalone solution to measure anode and cathode potentials.

### 2.6 Comparison

**Table** **1** presents a summary of the applicability, advantages, and disadvantages of each setup, along with suggestions for further improvement and verification experiments specific to each setup.

**Table 1:** Comparison of five integration methods for reference electrodes. Recommendations for application (cell potential deconvolution (U/I) or EIS), advantages, disadvantages and suggested verification methods and improvements for each setup.

	edge‐type	wire (inactive)	wire (active)	salt‐bridge	Electrolyte
Suitable for U/I	✓	✓	x	✓	x
Suitable EIS	✓	✓	✓✓	x	x
Advantages	‐Reliable ‐Simple	‐Simple	‐Simple ‐Artifact free EIS spectra	‐Allows measuring local potentials	
Disadvantages	‐Alignment control required ‐ Restricted to GDEs	‐Alignment control required ‐Restricted to GDEs ‐Drifting reference potential	‐Drifting reference potential ‐Affects cell performance ‐Working point affects reference potential	‐Bad reproducibility due to delicate GDL impregnation	‐Does not fully split anode and cathode contribution due to inadequate contact point
Suggested verification	Verify alignment by using two REs	Verify alignment by using two REs Show stability of reference potential	Compare cell performance with/without reference	Verify reproducibility	
Suggested improvement		alternative wire RE	alternative wire RE Reduced footprint of RE Investigate influence of cell operation on RE potential	Optimize impregnation (amount / dilution of ionomer solution)	

We identify the edge‐type setup, wire (inactive) setup, and salt‐bridge setup as viable options for deconvoluting cell potential. The edge‐type and wire (inactive) setups offer straightforward implementation but require precise electrode alignment to prevent measurement errors. To address this, we propose employing two reference electrodes positioned on opposing sides of the cell to demonstrate adequate alignment control. Additionally, for setups utilizing a wire reference electrode, we recommend reporting the reference potential before and after testing to showcase its stability.

The salt‐bridge setup requires further optimization of the impregnation of the gas diffusion layer to enhance experimental reproducibility. However, exploring this further is worthwhile, because localized measurement of potentials could significantly contribute to the understanding of CO_2_ electrolyzers.

Conversely, the wire (active) setup and electrolyte setup are not well suited for accurate cell potential deconvolution. The wire (active) setup affects the cell performance negatively and demands a deeper understanding on how different species in the electrolyzer affect the reference potential. However, it may be feasible to use this setup to qualitatively observe the electrode potentials over time to attribute the source of degradation, if the potential of the reference wire is frequently checked. The electrolyte setup fails to correctly split anode and cathode contributions due to an inadequate contact point. A significant part of the anode losses are measured between cathode and reference electrode in this setup.

Edge‐type setup and both wire setups are generally suited for impedance spectroscopy. However, edge‐type and wire (inactive) setup require sufficient electrode alignment. We consider the wire (active) setup the most promising for impedance spectroscopy, because it is the only setup in which the data is not obscured by artifacts at high frequencies.

Overall, the edge‐type and wire setups (both active and inactive) are deemed more practical for impedance measurements compared to the salt‐bridge setup, due to the challenges in assembling the salt‐bridge.

## 3 Conclusion

In this study, we provide a comprehensive guide to the integration of reference electrodes into a zero‐gap CO_2_ electrolyzer. We conduct direct experimental comparison of five different setups, emphasizing potential sources of error for each. Additionally, we employ numerical simulations to validate and elucidate these sources of error. Given the difficulty in obtaining error‐free data from integrated reference electrodes, it is crucial to thoroughly verify each experimental setup before employing it in electrolysis cell investigations.

If the alignment of the electrodes can be controlled sufficiently (e.g., by utilizing gas diffusion electrodes), utilizing a membrane strip connected to an external reference electrode or a wire reference electrode to contact the cell within the inactive area offers a straightforward solution to obtain accurate data. Using a membrane strip is preferable, because stability of the reference electrode is less of a concern in this setup.

Sandwiching a wire reference electrode between two pieces of membrane is a promising strategy to measure artifact free impedance spectra. However, the wire can impact the performance of the electrolysis cell and thus create data, which is not representative of the tested cell. Using a salt‐bridge reference electrode may be used for localized potential measurements, however, the reproducibility needs to be enhanced. We suggest further optimization of both setups, as they could significantly contribute to our understanding of CO_2_ electrolyzers, if these challenges are addressed.

As a concluding remark, we would like to emphasize the importance of validating data obtained from setups with an integrated reference electrode. Many sources of error can be difficult to detect yet lead to erroneous conclusions about the system. Ensuring, that a setup is capable to generate accurate data is a vital prerequisite for gaining a deeper understanding of CO_2_ electrolyzers.

## 4 Experimental Section

### Materials

IrO_2_ nanopowder (Iridium(IV) oxide, Premion) and silver nanoparticles (APS 20–40 nm, 99.9% (metal basis)) were obtained from Alfa Aesar. Nafion dispersion D520 was purchased from Ion Power. PTFE dispersion (60 wt% in H_2_O), potassium hydroxide pellets (ACS reagens, 85%), potassium chloride, isopropyl alcohol (ACS reagent), silver nitrate (ACS reagent, >99.0%) and silver wire (0.1 mm diam., 99.9% metal basis) were purchased from Sigma Aldrich. Nickel felts (200 µm, BEKIPOR 2NI06‐0,20) were acquired from Bekaert. Freudenberg H23C6 gas diffusion layers were purchased from QuinTech e.K. The Aemion+ anion exchange membrane (AF2‐HNN8‐50‐X) and Aemion+ ionmer (AF2‐HNN8) were purchased from Ionomr Inc. PTFE sheets of various thicknesses were purchased from Böhme Kunststofftechnik. Fluoropolymer dispersion (Teflon FEPD 121) was purchased from Fuelcell Store and Aquivion (D98‐25BS, 25 % in water) from Sigma Aldrich. Hydrochloric acid (36% w/w aq. soln.) was purchased from thermo scientific. Kapton tape (64 µm x 6 mm) was purchased from Conrad Electronic. Fuel Cell ePTFE III filter membrane was obtained from Donaldson. Deionized water was used and all chemicals were used without further purification.

### Electrode Fabrication

Anode catalyst layers were fabricated using an atomized ultrasonic spray coater (Sono‐Cell SNR300, Sonaer). The ink consisted of Premion IrO_2_ (0.4 g), H_2_O (11.31 g), isopropyl alcohol (9.46 g) and Nafion dispersion D520 (0.62 g). The ink was sonicated with an ultrasonic horn for 30 min prior to coating. An ultrasonic nozzle with 60 kHz and a power of 6 W and a flow rate of 0.2 mL min^−1^ were used. The substrate was heated to 40 °C. The catalyst material was applied to a 4 cm^2^ nickel felt to a loading of 0.15 mg cm^−2^ for all setups but the salt‐bridge setup. For the latter, the catalyst was directly applied to the Aemion+ membrane. The thinner membranes for the wire reference electrode (active) setup were fabricated by pouring a freshly filtered solution of Aemion+ (7 wt% in MeOH with 2 wt% DMAc) on a PTFE sheet and distributing it with a knife coater (BYK Instruments). A wet film thickness of 600 µm led to a membrane with a thickness of ≈ 25 µm.

Cathode catalyst layers were also fabricated by ultrasonic spray coating. The ink consisted of silver nanoparticles (0.25 g), H_2_O (17.59 g), isopropyl alcohol (13.83 g) and 5 wt% PTFE dispersion (1.67 g, diluted from 60 wt% stock solution). Fabrication parameters were identical to the anode. The catalyst material was applied to a 4 cm^2^ Freudenberg H23C6 gas diffusion layer to a loading of 0.1 mg cm^−2^ for all setups but the salt‐bridge setup. For the latter, the catalyst was directly applied to the Aemion+ membrane.

### Wire Reference Electrode Fabrication

The fabrication procedure for the wire reference electrodes was adapted from Hansen et al.^[^ ^8^ ^]^ 40 mm of silver wire were cleaned in H_2_O and 1 м KOH in an ultrasonic bath for 3 min each. The wire was reduced in 100 mL of 100 mм KCl and 100 mм HCl solution at −0.5 V versus saturated calomel electrode for 60 s. Silver chloride was formed by electrochemical conversion at 0.2 V versus saturated calomel electrode until a surface charge of 1 C/cm^2^ was reached, resulting in a nominal AgCl thickness of 3 µm.^[^ ^38^ ^]^ The wires were dipped in a 1:2.2 w/w dispersion of FEPD fluoropolymer and Aquivion for 10 min and dried at air for 10 min. It was then cured in the oven at 265 °C with 2 h of heating, curing and cooling, respectively. The wires were then stored in a 1 м KCl and 32 mм AgNO_3_ solution for 24 h and rinsed with water before testing.

### Salt‐Bridge Fabrication

A PTFE capillary (inner diam. 1.0 mm, outer diam. 1.6 mm, BOLA) was used for the salt‐bridge. A piece of Fuel Cell ePTFE III filter membrane was inserted in one end for about 5 mm. The ePTFE was then soaked with 2.5 wt% Aemion+ solution in 10:1 methanol:H_2_O, by drawing the ionmer solution through the capillary with a syringe, to encapsulate the end of the capillary. The ionmer was dried in an oven at 80 °C. After mounting the salt‐bridge in the cell fixture, the capillary was filled with 1 м KOH by using a needle from the backside.

### Impregnation of Gas Diffusion Layer

The gas diffusion layer was placed with the microporous layer facing down on a contraption build from a Falcon tube (see Figure S18, Supporting Information) to apply a vacuum to its bottom side. 10 µL of 0.25 wt% Aemion+ solution in 10:1 methanol:H_2_O was then placed on the gas diffusion layer and dried at air. This procedure was performed to a total of three times.

### Electrochemical Characterization

Membranes were stored in 1 м KOH for 24 h before testing and gas diffusion electrode were placed with the catalyst side in 1 м KOH for 15 min. A serpentine flow field (titanium grade 2) was used at the cathode side and a parallel flow field at the anode (stainless steel). The utilized gaskets depended on the setup, as described in the supplementary information. The fixture was closed with 8 bolts, tightened with a torque of 6 Nm.

The CO_2_ flow rate at the cathode was controlled to 95 mL min^−1^ with a mass flow controller (EL‐FLOW Prestige FG‐201CV, Bronkhorst). A bubbler at room temperature was used for gas humidification. The cell was operated at 50 °C and 0.1 м KOH was preheated to 50 °C and used as anolyte. When testing in the edge‐type setup with two reference electrodes, the reference electrode setup got in the way of the temperature control system. Thus, these experiments were performed at 30 °C. An additional experiment using only one reference electrode shows, that the difference in cell performance at the different temperatures was reasonably small (see Figure S21, Supporting Information).

Electrochemical measurements were performed with a BioLogic VSP‐300 potentiostat. For the start‐up procedure, the cell was operated at 2.5 mA cm^−2^ for 30 s, then at 1.9 V for 50 s. Two linear sweep voltammetries were performed from 0 V to 3.5 V with 50 mV s^−1^. For polarization curves, constant current steps of 12.5, 25, 50, 100, 200, 300, and 400 mA cm^−2^ were held for 10 min each. The gas from the cathode outlet was analyzed with an on‐line gas chromatograph (Agilent micro GC 990) after 9 min of each current step. Galvanostatic electrochemical impedance spectroscopy measurements with an amplitude equal to 1/20 of the constant current in a frequency range of 200 kHz to 1 kHz was performed and both working and counter electrode signals were recorded at each current step.

Galvanostatic electrochemical impedance spectra were recorded at 200 mA cm^−2^ with an amplitude equal to 1/10 of the current from 1 MHz to 100 mHz. Impedance spectra were measured individually between anode‐cathode, anode‐reference and reference‐cathode, with the potentiostats sense cable 1 connected to the more positive (first) electrode and sense cable 2 connected to the negative (second) electrode.

The Faradaic efficiency was calculated as $FE_{\mathrm{CO}}=\;z\;x_{\mathrm{CO}}\;F\;\frac{p}{R\;T}\frac{{\dot{V}}_{\mathrm{out}}}{I}100\%$, where *z* was the number of electrons needed to form CO from CO_2_, x_CO_ was the mole fraction of CO measured by the GC, Faraday's constant *F*, pressure *p*, ideal gas constant *R*, temperature *T*, product gas flow rate *V̇*_out_ as measured with a mass flow meter (EL‐FLOW Select F‐111B, Bronkhorst) and current *I*.

### Reporting of Potentials

All experimental results were reported as measured versus Hg/HgO (0.1 м KOH). For reference, the standard electrode potentials of oxygen evolution reaction (OER) and carbon monoxide evolution reaction (COER), as determined in Section S2 (Supporting Information). Simulation results were reported against reversible hydrogen electrode (RHE).

### Simulation

Finite element simulations were performed using COMSOL Multiphysics 5.4. For simplicity, a 2D simulation domain was used to simulate the ionic potential *Φ* throughout the membrane. Laplace's equation (∇^2^ *Φ*  =  0) was used as governing equation. The kinetics of both electrodes were implemented as boundary condition by Butler‐Volmer equation. Representative kinetic parameters for the electrodes were adapted from literature reports,^[^ ^39^, ^40^ ^]^ but do not precisely represent the electrodes used in the experiments. Thus, simulations were exclusively used for qualitative understanding. The electrical potential was set to the cell voltage at the anode and to 0 V at the cathode. Impedance spectra were simulated in a frequency range from 100 kHz to 10 mHz. All parameters used for the simulation and adaptations for simulated special cases were described in the supplementary information.

## Conflict of Interest

The authors declare no conflict of interest.

## Supporting information

Supporting Information

## Data Availability

The data that support the findings of this study are available from the corresponding author upon reasonable request.
