# Supplementary material for: Reference Electrode Types for Zero‐Gap CO2 Electrolyzers: Benefits and Limitations
Source: Adv Sci (Weinh). 2024 Jun 25;11(32):2402095. doi: 10.1002/advs.202402095 (PMC11348192; doi:10.1002/advs.202402095)
Supplement: Supplementary file 1 — Supporting Information [file ADVS-11-2402095-s001.pdf]

ADVANCED SCIENCE
Open Access

Supporting Information

for *Adv. Sci.*, DOI 10.1002/advs.202402095

Reference Electrode Types for Zero-Gap CO_2 Electrolyzers: Benefits and Limitations

*Luca Bohn, Jochen Kieninger, Stefan J. Rupitsch, Carolin Klose, Severin Vierrath and Joey Disch**

Supporting Information

Reference electrode types for zero-gap CO_2 electrolyzers: benefits and limitations

Authors:

Luca Bohn[1,2], Jochen Kieninger[3], Stefan J. Rupitsch[3], Carolin Klose[1], Severin Vierrath[1,2] and Joey Disch[1,2*]

Affiliations:

[1] Electrochemical Energy Systems, IMTEK – Department of Microsystems Engineering, University of Freiburg, Georges-Koehler-Allee 103, 79110 Freiburg, Germany

[2] FIT – Freiburg Center for Interactive Materials and Bioinspired Technologies, University of Freiburg, Georges-Koehler-Allee 105, 79110 Freiburg, Germany

[3] Laboratory for Electrical Instrumentation and Embedded Systems, IMTEK – Department of Microsystems Engineering, University of Freiburg, Georges-Koehler-Allee 106, 79110 Freiburg, Germany

*Corresponding author: Joey.Disch@imtek.uni-freiburg.de

Figure S1: Reproducibility of measurements in two electrode setup. (…) 2
Figure S2: Edge-type reference electrode setup. (…) 2
Figure S3: Full impedance spectra for all setups (…) 3
Figure S4: Simulated electrode potentials with small misalignment (…) 5
Figure S5: Electrode potentials measured with a misalignment of 1 mm and iR-compensation in the edge-type setup. 6
Figure S6: Simulation of the effect of iR-compensation. (…) 7
Figure S7: Practical remarks on the edge-type reference electrode setup (…) 8
Figure S8: Simulated effect of introducing a contact point at the membrane strip. (…) 8
Figure S9: Typical catalyst coated membrane. (…) 9
Figure S10: Inactive wire reference electrode setup. Setup scheme with utilized gaskets. 10
Figure S11: Ex-situ validation of the silver-silverchloride wire electrode. (…) 10
Figure S12: Wire reference electrode potential after each fabrication step. (…) 11
Figure S13: Active wire reference electrode setup. Setup scheme with utilized gaskets. 11
Figure S14: Simulated effect of electrode misalignment on measured electrode potentials and impedance spectra in all setups (…) 12
Figure S15: Comparison signal noisiness in the active and inactive wire reference electrode setup during polarization experiments. 13
Figure S16: Salt-bridge reference electrode setup. Setup scheme with utilized gaskets. 13
Figure S17: Simulation of measured potentials with a gap in the cathode catalyst layer. 14

Figure S18: Setup used to achieve full impregnation of the gas diffusion layer. (…) 14
Figure S19: Simulation of results obtained from an over-impregnated gas diffusion layer in the salt-bridge setup. (…) 15
Figure S20: Electrolyte reference electrode setup. Setup scheme with utilized gaskets and housing for the reference electrode. 16
Figure S21: Comparison of electrode potentials measured in edge-type reference electrode setup at 50 °C and 30 °C. 17

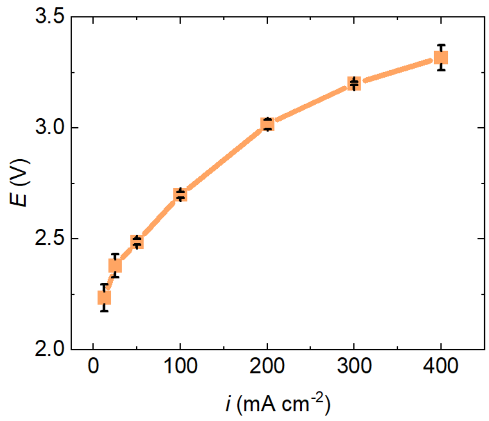

Figure S1: Reproducibility of measurements in two-electrode setup. The measurement shows the mean of three independent zero-gap cells, the error bars show the standard deviation, which is 56 mV at 400 mA cm^{-2}.

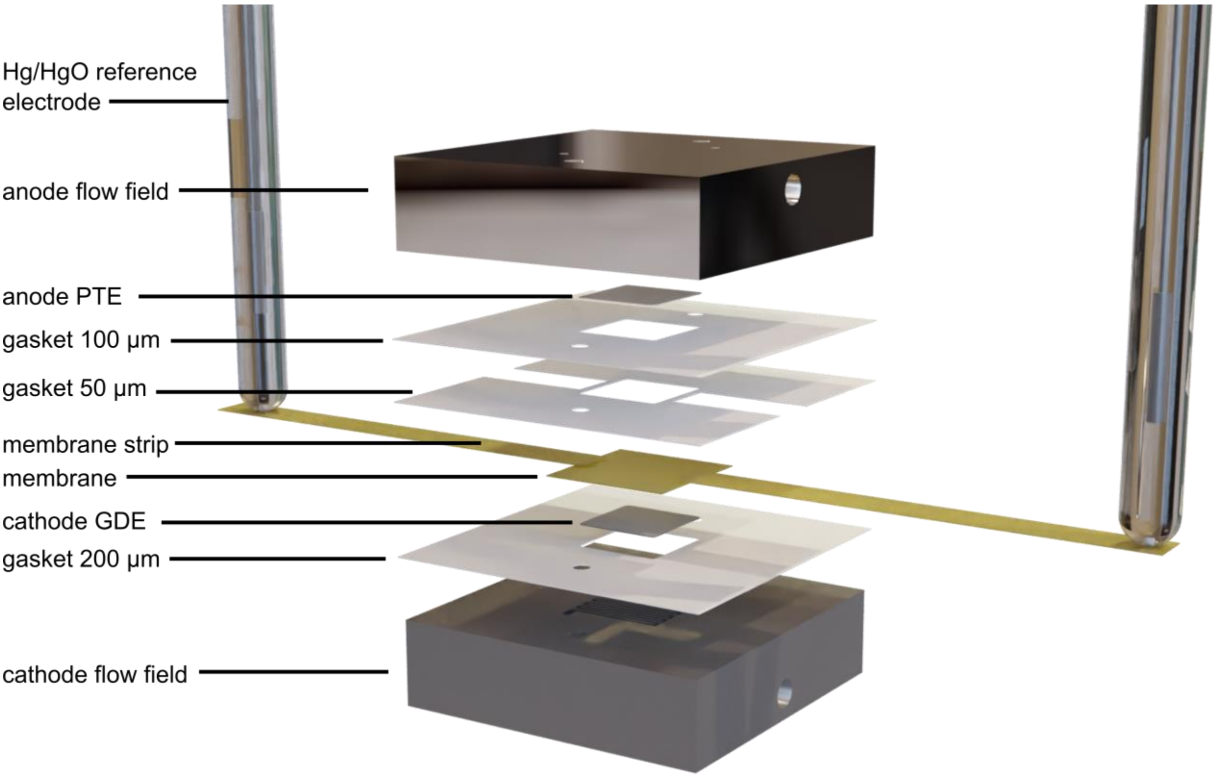

Figure S2: Edge-type reference electrode setup. Setup scheme with utilized gaskets. The membrane strips were humidified with 1 M KOH within a cavity in the custom fixture.

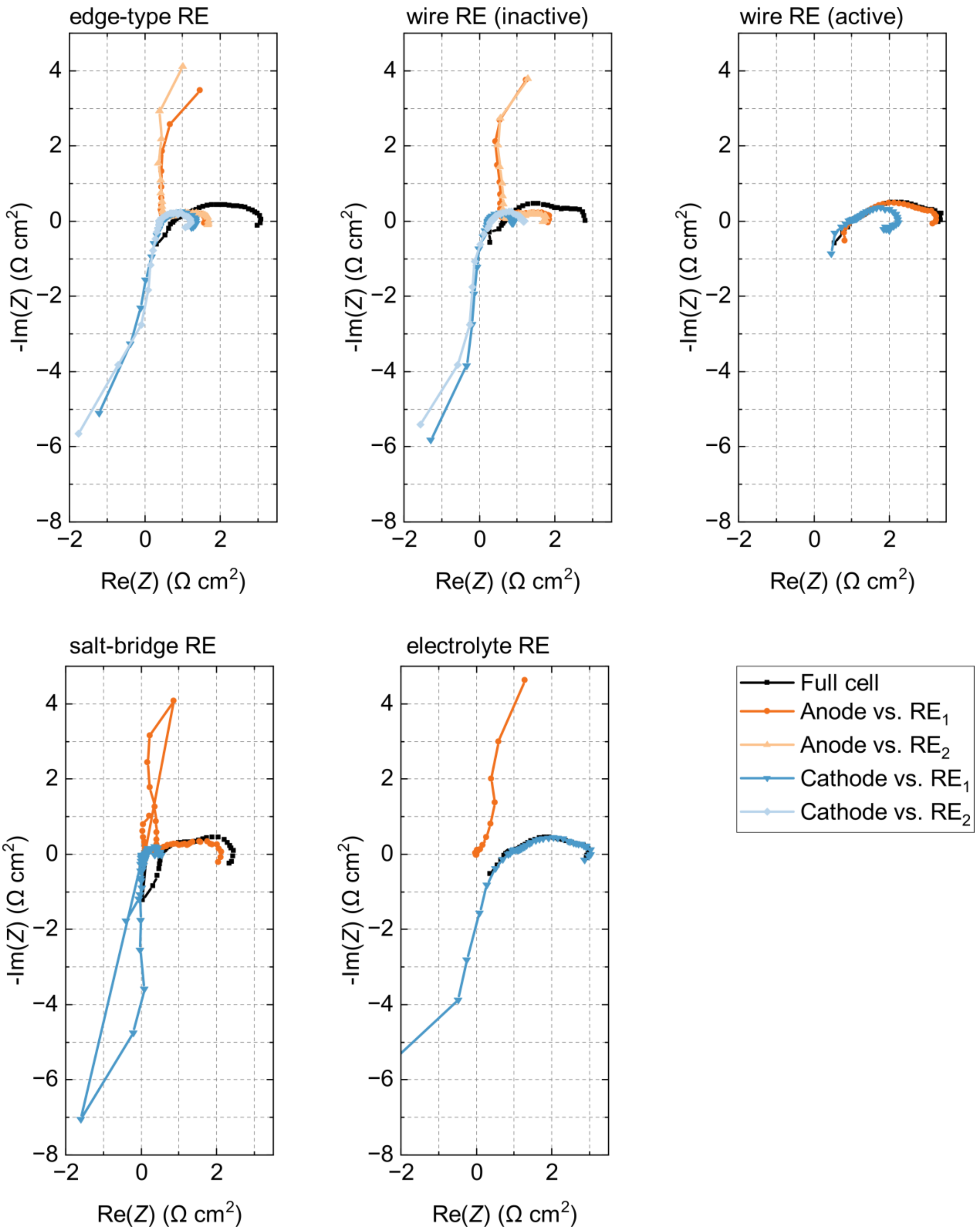

Figure S3: Full impedance spectra for all setups: *Artifacts in electrochemical impedance spectra at high frequencies.*

Section S1: Base case simulation of electrode potentials, potential distribution and impedance spectra.

All simulations were performed using COMSOL Multiphysics 5.4. The simulation domain comprises a cross-section of the membrane, as indicated in Figure 1 in the main document, with anode and cathode implemented as boundary conditions. The potential distribution within the membrane is described via Laplace's equation (Eq. 1) with the potential Φ versus the electrical potential at the cathode.

$$\nabla^2\Phi = 0 \tag{1}$$

The current i in the electrolyte is determined by Ohm's law (Eq. 2) with an electrolyte conductivity σ of 6.5 S m^{-1}.

$$i = -\sigma\nabla\Phi = 0 \tag{2}$$

To describe both electrode's kinetics, Butler-Volmer Equation is used (Eq. 3-4)

$$i_{\perp,a} = i_{0,a}\left(exp\left[\frac{\alpha_a n_a F\,\eta_a}{RT}\right] - exp\left[\frac{-(1-\alpha_a)\,n_a F\,\eta_a}{RT}\right]\right) \tag{3}$$

$$i_{\perp,c} = i_{0,c}\left(exp\left[\frac{(1-\alpha_c) n_c F\,\eta_c}{RT}\right] - exp\left[\frac{-\alpha_c\,n_c F\,\eta_c}{RT}\right]\right) \tag{4}$$

with vertical current density $i_\perp$ at anode an cathode, current exchange density i_0, symmetry coefficients α_a and α_c for each half cell's reaction, number of electrons n transferred per reaction step, faraday's constant F, ideal gas constant R, electrolyzer temperature T = 323.15 K and overpotential η at each electrode. The reactions at each electrode are assumed to be purely CO evolution reaction (Eq. 5) at the cathode and oxygen evolution reaction (Eq. 6) at the anode, taking place at -0.11 V vs. RHE and 1.23 V vs. RHE, respectively.

$$CO_2 + H_2O + 2\ e^- \rightarrow CO + 2\ OH^- \tag{5}$$

$$4\ OH^- \rightarrow O_2 + 2\ H_2O + 4\ e^- \tag{6}$$

Thus, n_c = 2 and n_a = 4. At all other boundaries, a zero-current boundary condition is assumed. The electrical potentials of the anode and cathode are set to the cell voltage and to zero, respectively. The overpotential at each electrode can be expressed in terms of the ionic potential Φ_{ion} at that electrode (Eq. 7-8).

$$\eta_c = \phi_{ion,c} + E_{0,c} \tag{7}$$

$$\eta_a = E_{cell} - \phi_{ion,a} - E_{0,a} \tag{8}$$

Kinetic parameters for Ag and IrO_2 based electrodes were obtained from literature data (see Table S1).[1, 2]

Table S1: Kinetic and geometric simulation parameters

Parameter	Value
$i_{0,a}$	4.0 × 10^{-10} A cm^{-2}
α_a	0.40
$i_{0,c}$	2.32 × 10^{-4} A cm^{-2}
α_c	0.138
w	30 mm
w_{el}	20 mm
t	50 µm

Electrode potentials were simulated using the secondary current distribution module and a parametric sweep of the cell voltage from 1.6 V to 2.5 V. For impedance spectra, a frequency domain perturbation study in the range of 10 mHz to 100 kHz was performed. Double-layer capacities C_{dl} of 300 mF cm^{-2} and 1 mF cm^{-2} were assumed for anode and cathode.

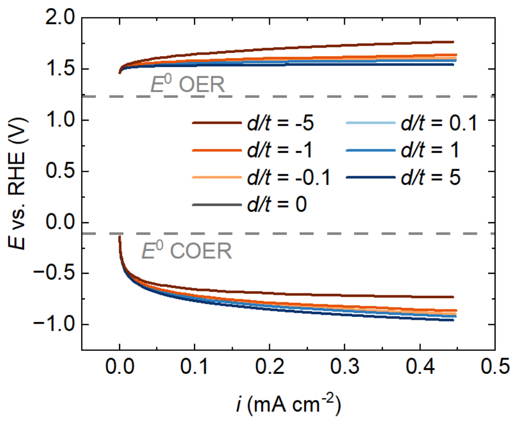

Figure S4: Simulated electrode potentials with small misalignment in the edge-type setup. The accuracy of the measurement increases when electrodes are placed accurately. In simulations, a misalignment in the range of -1 < *d/t* < 1 (less than 50 µm) leads to an error < 31 mV at 400 mA cm^{-2}. For -0.1 < *d/t* < 0.1 (less than 5 µm misalignment) the error is less than 3 mV. The required accuracy for the experiment depends on the application, however the simulation suggests that it is possible to control the alignment sufficiently. The accuracy should also be determined experimentally (e.g. by placing two reference electrodes on opposing sides of the cell and comparing the results), because the simulation results cannot be directly translated to experiments.

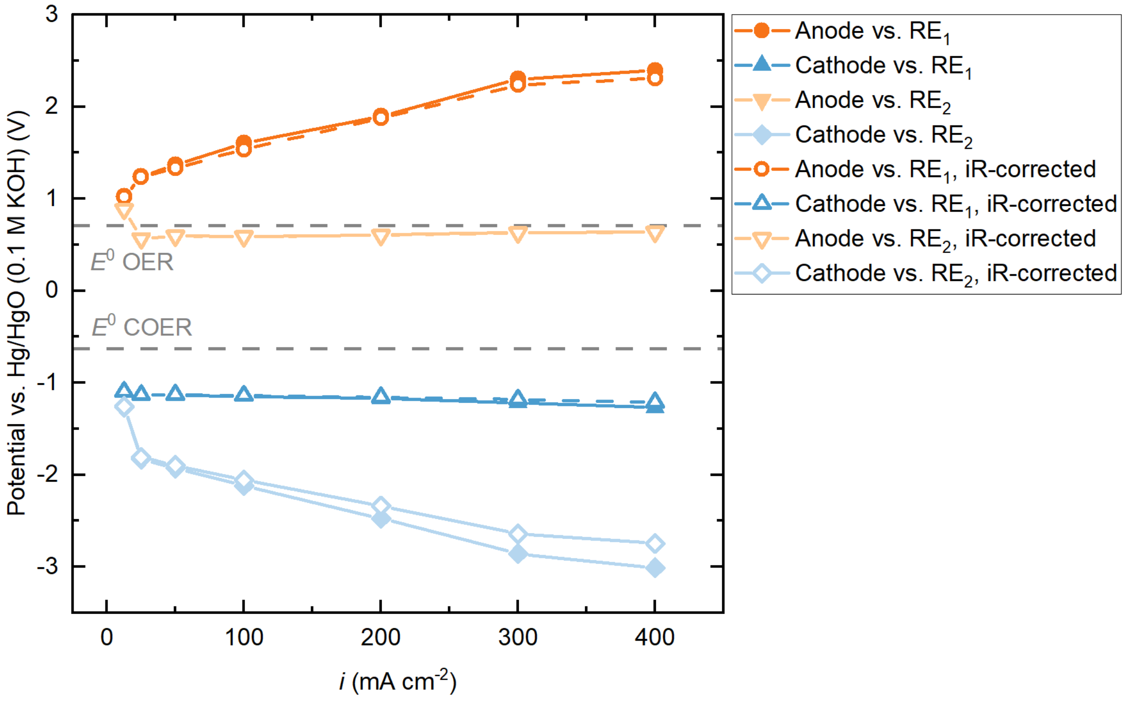

Figure S5: Electrode potentials measured with a misalignment of 1 mm and iR-compensation in the edge-type setup.

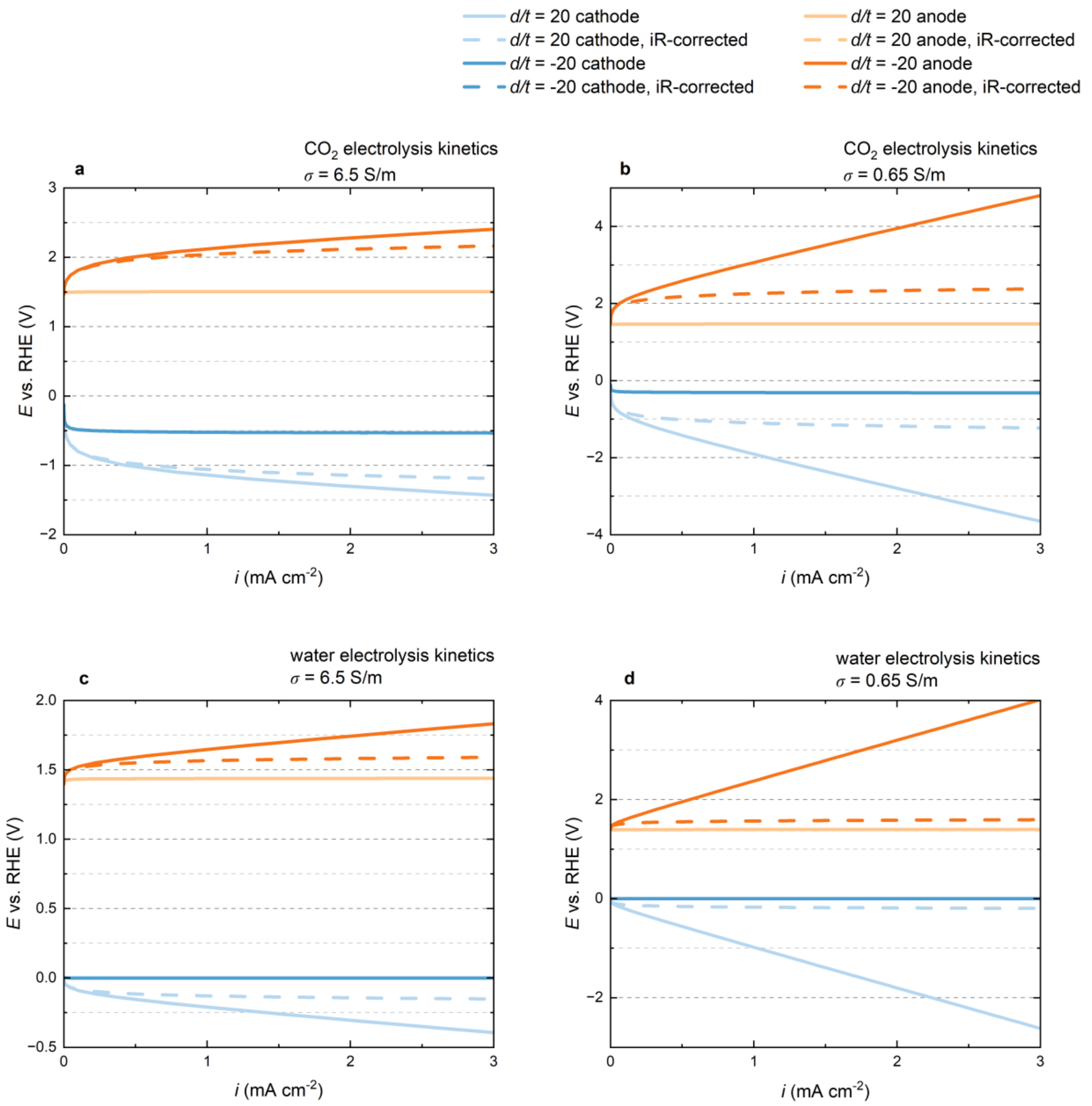

Figure S6: Simulation of the effect of iR-compensation. (a) In the base case, (b) with decreased membrane conductivity, (c) with faster electrode kinetics common for polymer electrolyte membrane water electrolysis[3] and (d) with faster kinetics and decreased membrane conductivity.

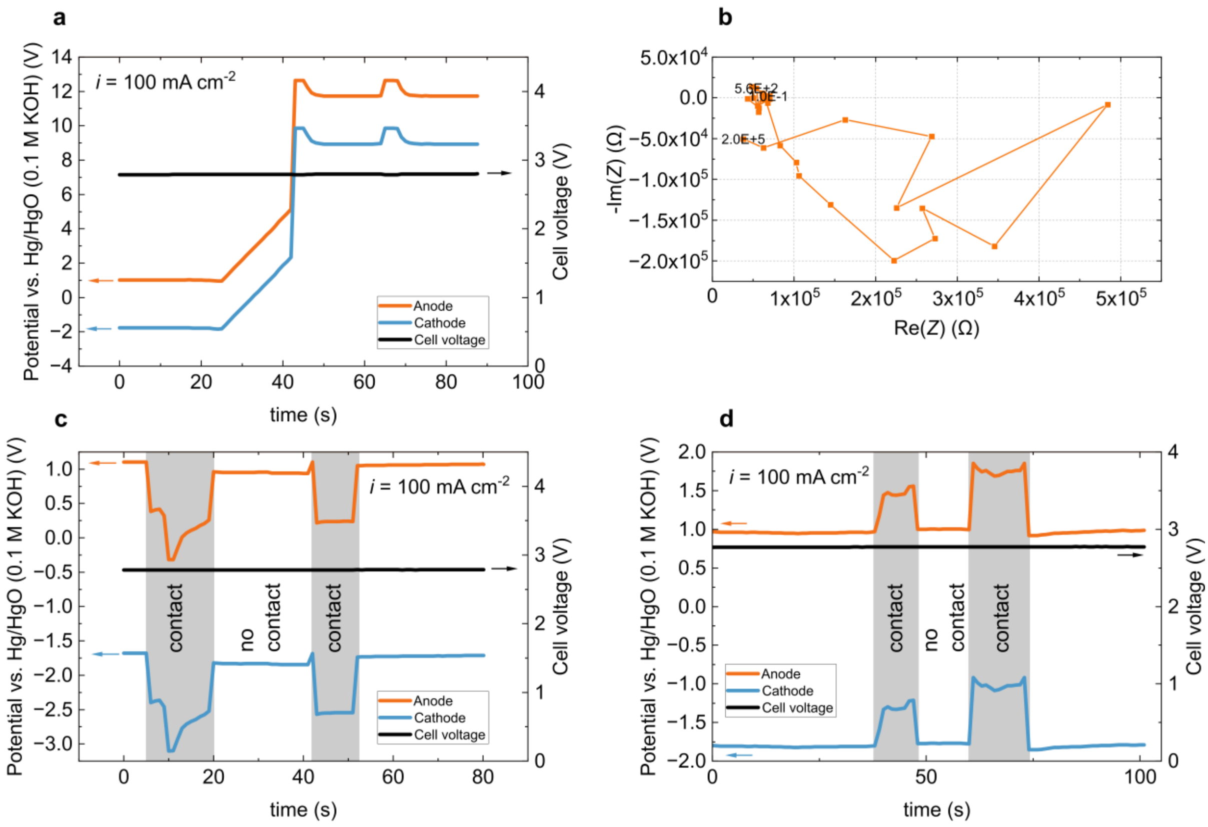

Figure S7: Practical remarks on the edge-type reference electrode setup: (a) Measured electrode potentials during intentional dry-out of the membrane strip. After 20 s under constant current operation, the KOH filled cavity in the fixture is drained, thus triggering dry-out of the membrane strip. As a consequence of insufficient ionic contact, the reference potential starts drifting, leading to a parallel drift in measured electrode potentials, while the cell voltage remains stable. (b) measurement of the impedance across a sufficiently humidified membrane strip, (c) measured electrode potentials if the strip is brought repeatedly into contact with the anode flow field or (d) the cathode flow field. Contact to the anode flow field leads to a shift to negative measurement values, contact to the cathode to positive values. The effect strongly affects the measured data, but is reversible, as the measured potentials return to their baseline, when the contact is broken.

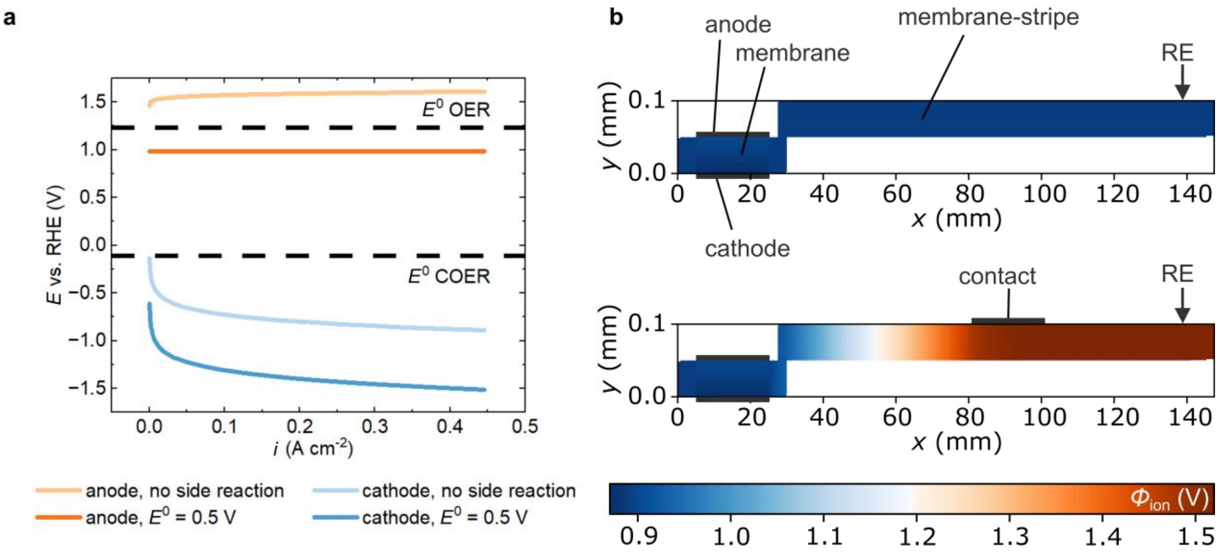

Figure S8: Simulated effect of introducing a contact point at the membrane strip. (a) Electrode potentials with and without contact and (b) potential distribution in membrane and strip without and with contact. For this simulation, the domain of the base case (see section S1) was extended by the membrane strip as sketched in Figure S6b. The contact was implemented as a less active electrode. Its

electrical potential was set to the cell voltage and $\boldsymbol{E_0}$ of 0.5 V vs. RHE, $\boldsymbol{i_0}$ of 4 × 10^{-18} A cm^{-2}, $\boldsymbol{n}$ equal to 4 and $\boldsymbol{\alpha_a}$ of 0.4 were assumed.

Section S2: Determination of standard electrode potentials E^0 for anode and cathode

The standard electrode potentials were determined as

$$E^0_{\text{OER vs Hg/HgO}} = 1.23\ \text{V} - \text{pH}\ \frac{\ln(10)\, R\, T}{F} - 0.157\ \text{V} = 0.711\ \text{V} \quad (9)$$

$$E^0_{\text{COER vs Hg/HgO}} = -0.11\ \text{V} - \text{pH}\ \frac{\ln(10)\, R\, T}{F} - 0.157\ \text{V} = -0.629\ \text{V} \quad (10)$$

with faraday's constant F, ideal gas constant R, electrolyzer temperature T = 323.15 K and pH equal to 13, corresponding to the utilized 0.1 M KOH anolyte. 0.157 V is the potential of the utilized Hg/HgO (0.1 M KOH) reference electrode vs. standard hydrogen electrode (SHE).[4]

Simulated values reported against reversible hydrogen electrode (RHE) relate by

$$E_{\text{RHE}} = E_{\text{SHE}} + \frac{\ln(10)\, RT}{F}\text{pH}$$

to SHE.[5]

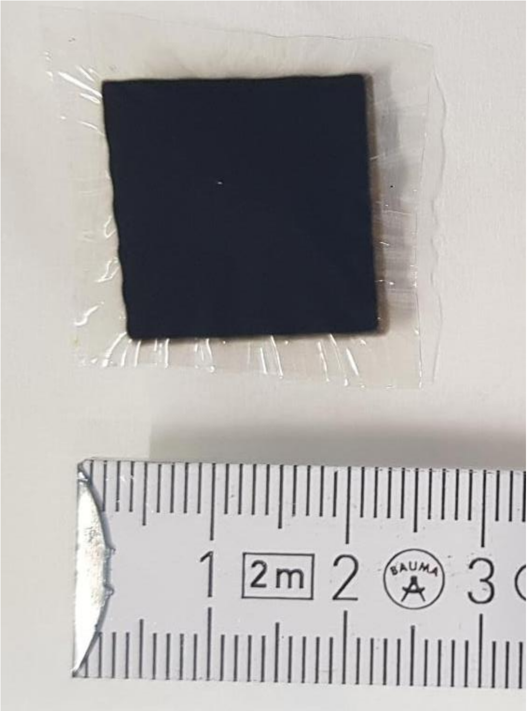

Figure S9: Typical catalyst coated membrane. Due to the membrane swelling during fabrication, both catalyst layers are displaced by roughly 1 mm. The cathode catalyst layer (grey) shows to a significant amount beyond the anode catalyst layer (black).

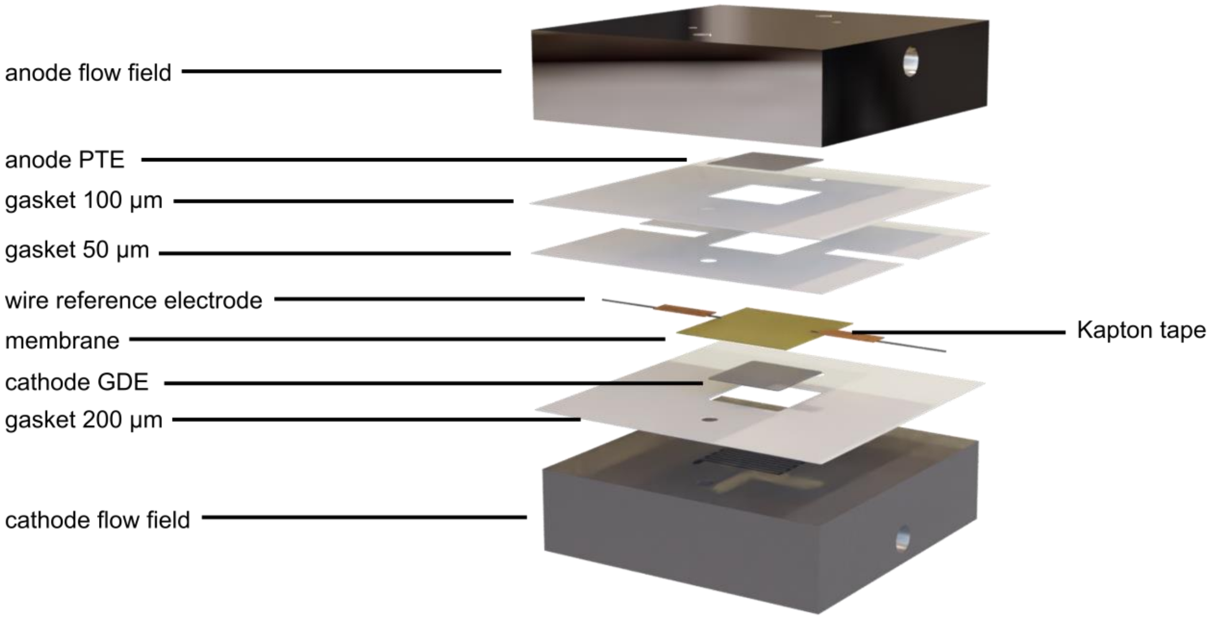

Figure S10: Inactive wire reference electrode setup. Setup scheme with utilized gaskets.

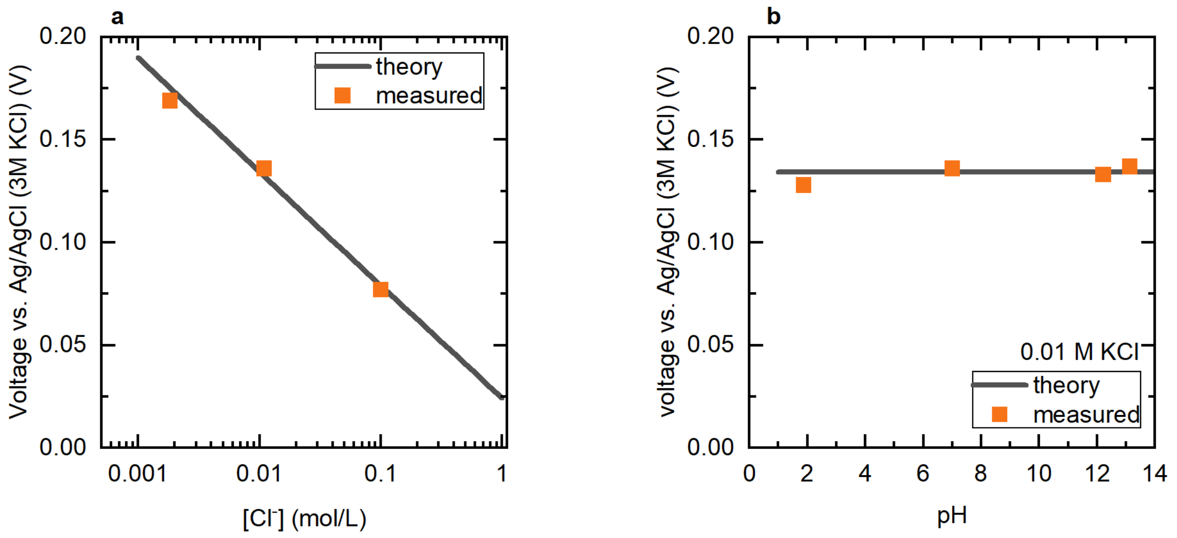

Figure S11: Ex-situ validation of the silver-silverchloride wire electrode. Comparison of the expected theoretical potential of a silver chloride electrode and the potential measured with the wire reference electrode in (a) KCl solutions with differing Cl^- ion concentration and (b) 0.01 M KCl solution at different pH values by adding H_2SO_4 or KOH.

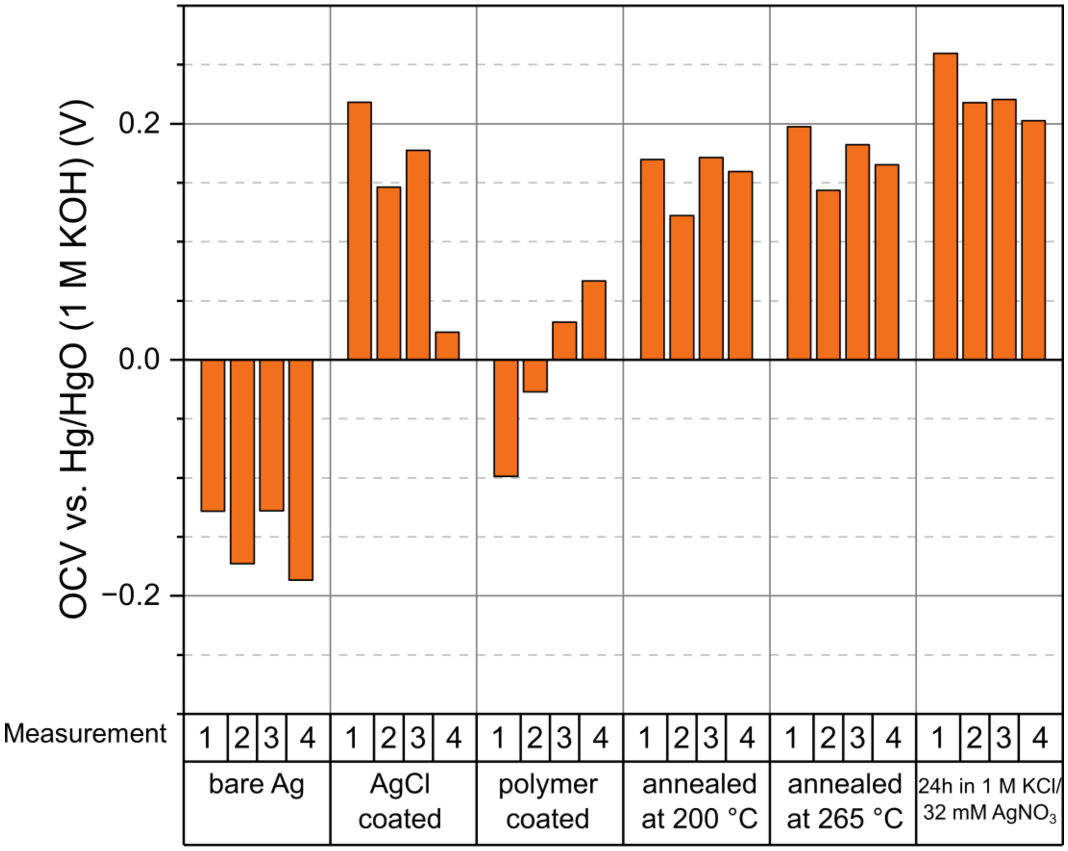

Figure S12: Wire reference electrode potential after each fabrication step. A sample wire taken after each fabrication step (bare Ag, AgCl coated, etc.) was placed within the same cell. The reference potential versus an external Hg/HgO electrode is measured (1) prior to all experiments, (2) after taking a polarization curve, (3) after 16 h resting and (4) after another polarization curve.

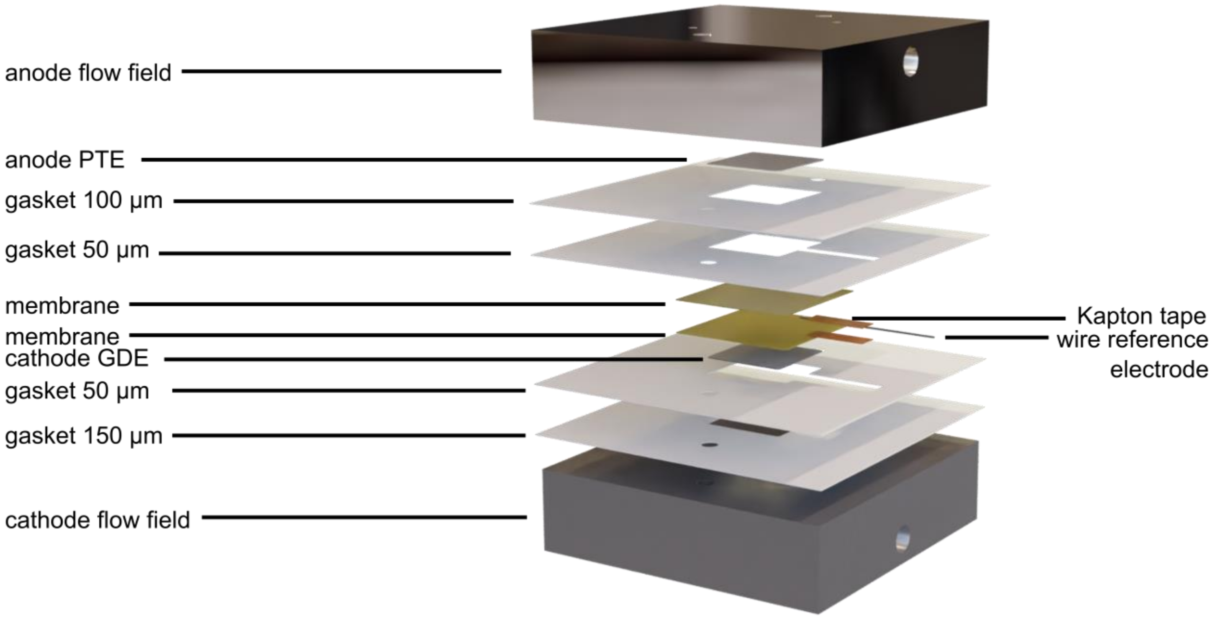

Figure S13: Active wire reference electrode setup. Setup scheme with utilized gaskets.

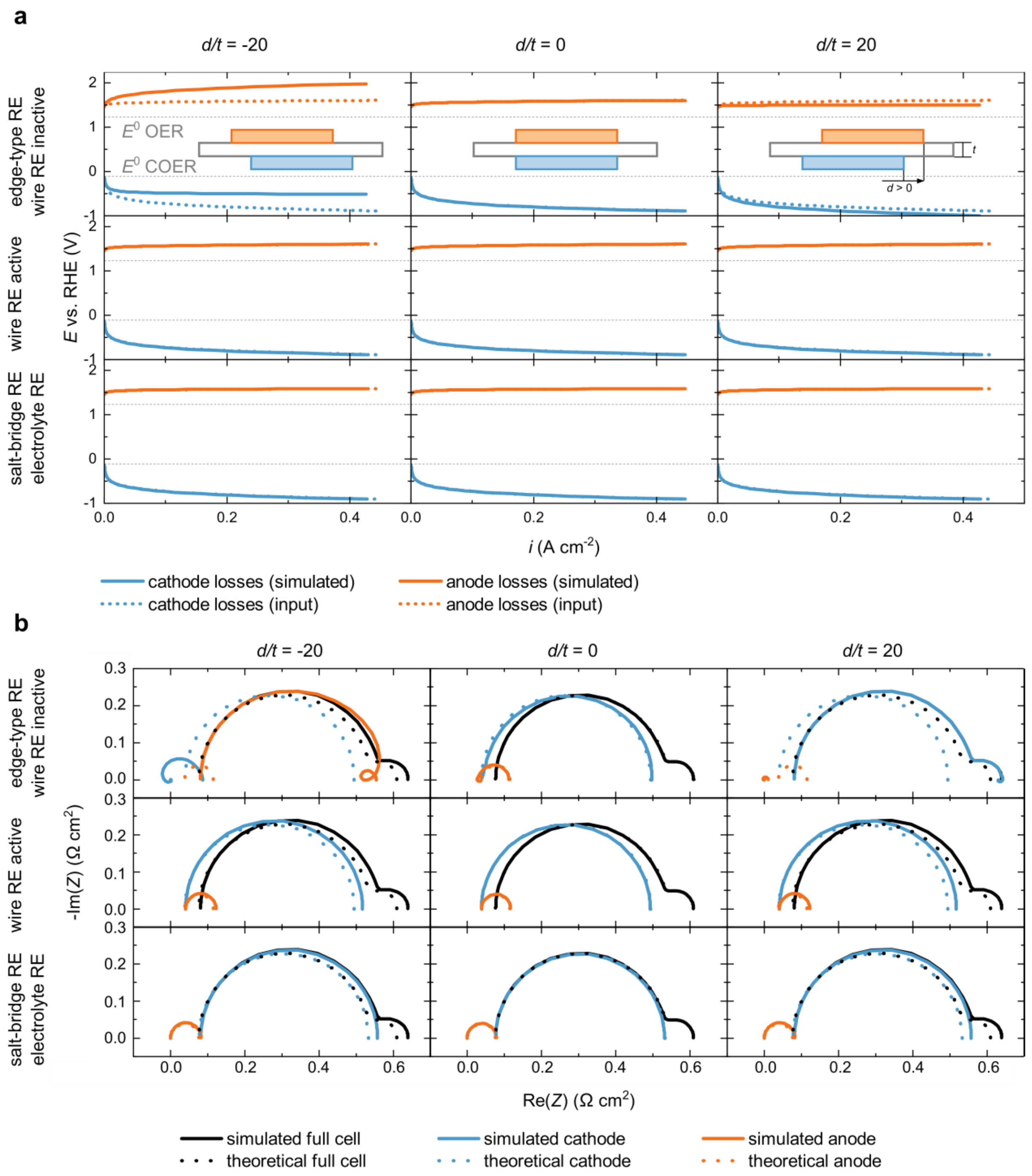

Figure S14: Simulated effect of electrode misalignment on measured electrode potentials and impedance spectra in all setups: (a) simulated electrode potentials and (b) simulated impedance spectra. All simulations in Figure S12 were performed as described for the base case in Section S1. The position of the reference electrode was changed from the inactive area, more than three membrane thicknesses from the electrodes (edge-type RE, wire RE (inactive)) to the middle of the active area (wire RE active) or right beneath the anode catalyst layer (salt-bridge RE, electrolyte RE). In the experiments, the contact of the salt-bridge RE is at the cathode side, thus to be more correct, an additional data set with the reference electrode beneath the cathode catalyst layer could be performed for this case. However, this is functionally identical to the third simulated case here, with only the HFR shifting from cathode to anode side.

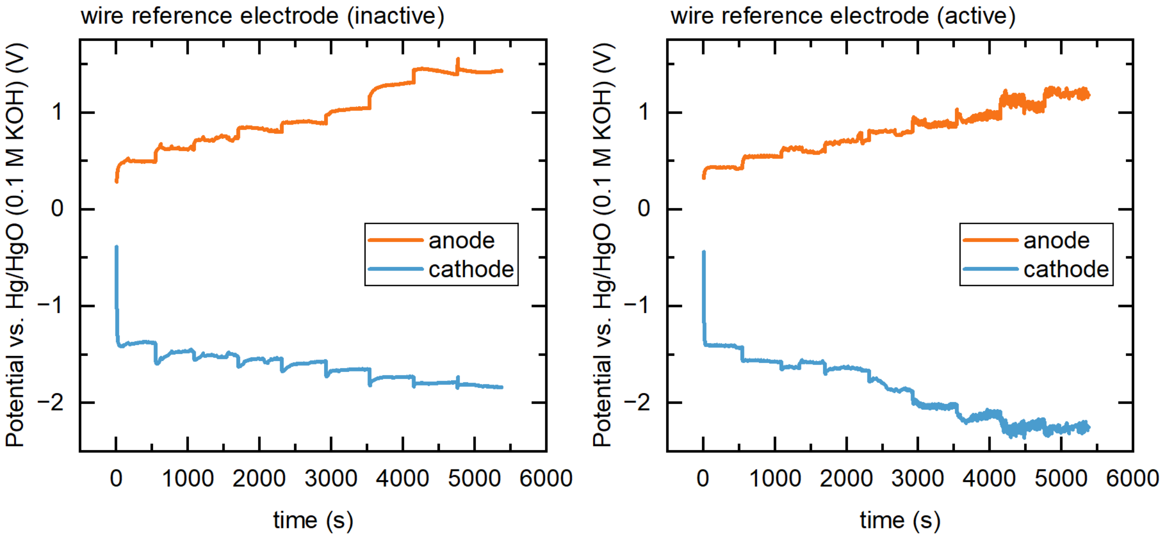

Figure S15: Comparison signal noisiness in the active and inactive wire reference electrode setup during polarization experiments.

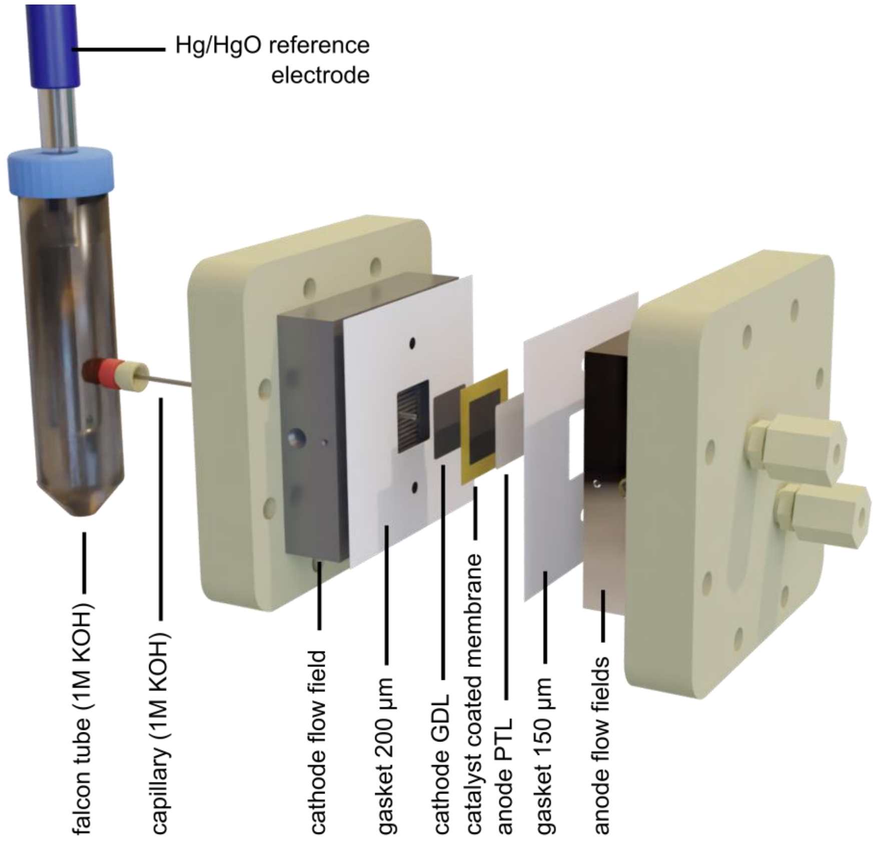

Figure S16: Salt-bridge reference electrode setup. Setup scheme with utilized gaskets.

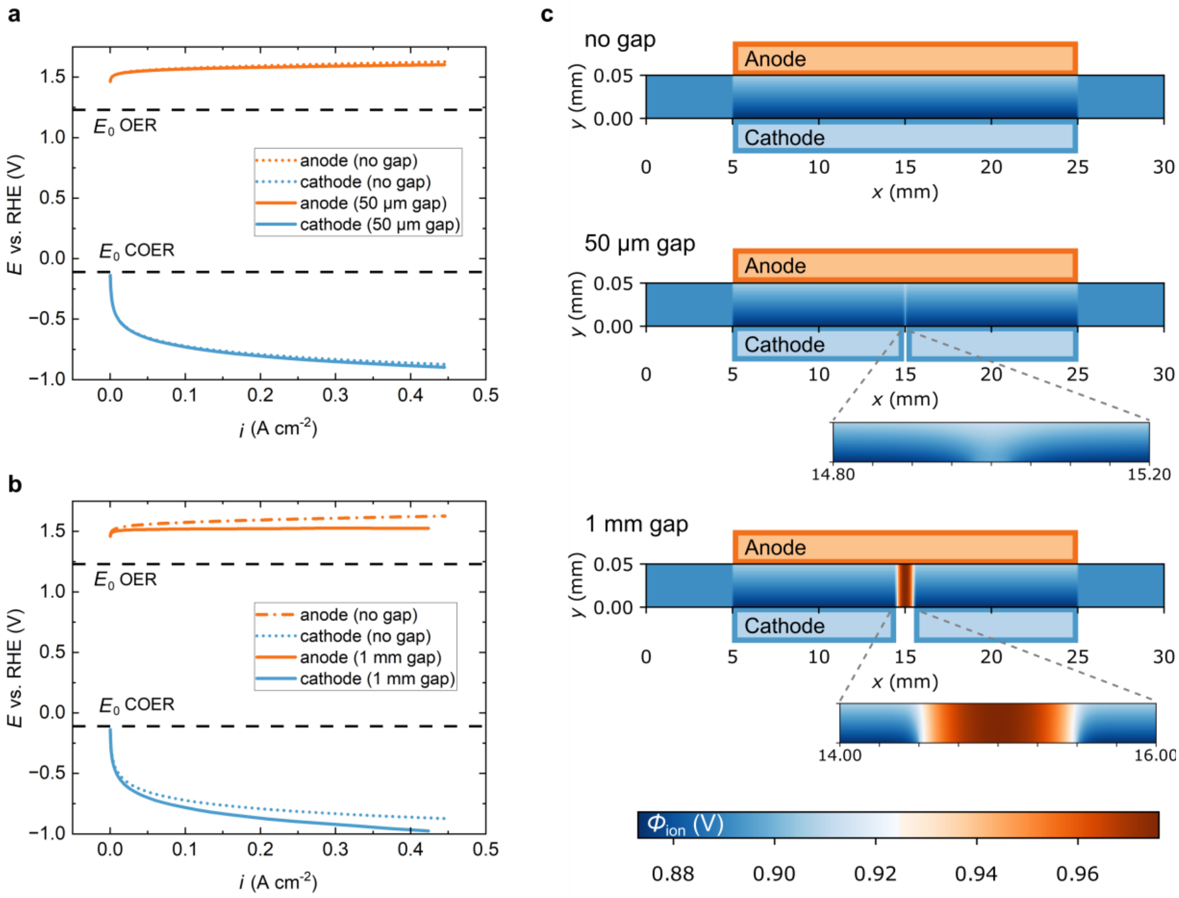

Figure S17: Simulation of measured potentials with a gap in the cathode catalyst layer. (a) Assuming a gap size of 50 µm or (b) 1 mm. (c) Potential distribution at a cell voltage of 2.5 V throughout the membrane without a gap and with a gap of 50 µm or 1 mm diameter.

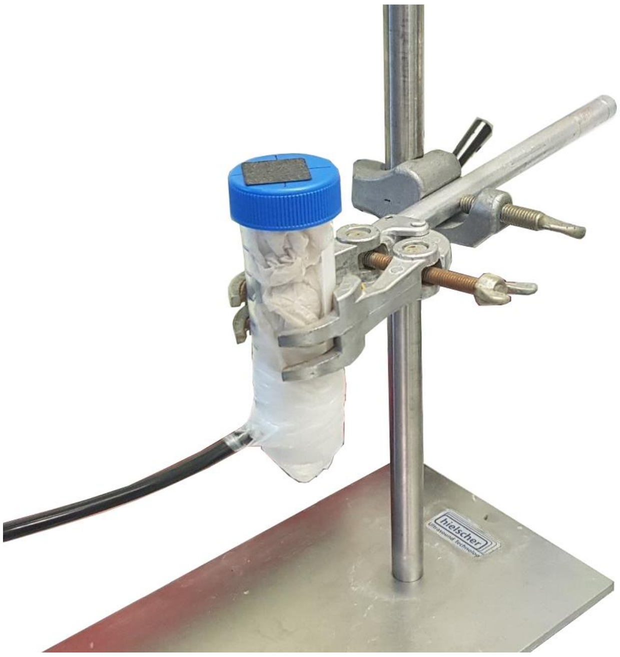

Figure S18: Setup used to achieve full impregnation of the gas diffusion layer. A falcon tube is used to apply a vacuum to the lower side of the gas diffusion layer.

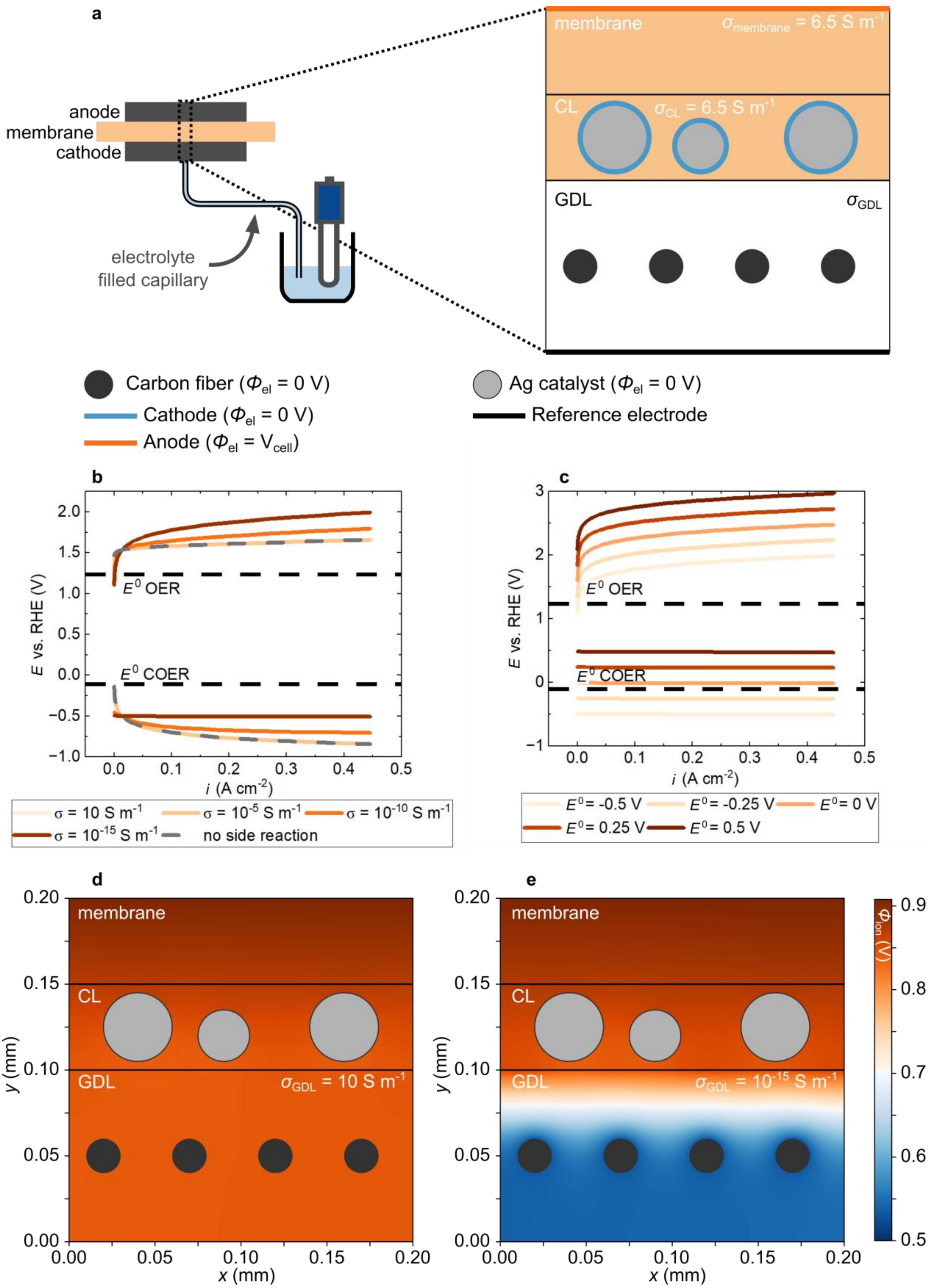

Figure S19: Simulation of results obtained from an over-impregnated gas diffusion layer in the salt-bridge setup. (a) Simulation scheme, (b) electrode potentials for different conductivities in the gas diffusion layer (i.e. different degrees of impregnation), (c) electrode potentials for different standard electrode potentials of the assumed side reaction at a conductivity of 10^{-15} S m^{-1} and (d) potential distribution at a cell voltage of 2.5 V with proper impregnation (high conductivity) and (e) insufficient impregnation (low conductivity).

Section S3: Information on the simulation in Figure S17

For this simulation, the simulation domain was changed according to Figure S17a. To obtain a similar full cell performance to previous simulations, i_0 of the cathode was corrected by a factor of 0.71.

An electrochemical side reaction at the position of the gas diffusion layer is assumed. This is only to demonstrate the effect of a side reaction and should not interpreted as a hypothesis for the process causing error in the measurements in CO_2 electrolysis cells. For the side reaction E_0 of -0.5 V vs. RHE, i_0 of 2.32 × 10^{-12} A cm^{-2}, n equal to 2 and α_c of 0.138 were assumed.

In Figure S17b, the conductivity within the gas diffusion layer due to the impregnation is varied. If the conductivity is sufficiently high, the measured electrode potentials are unaffected by the side reaction. However, if the contact is insufficient and the conductivity is low, the results become distorted and the cathode shows a constant signal. Figure S17c shows, that this constant potential is equal to the standard electrode potential assumed for the side reaction. This is, why the setup can be intentionally used to detect corrosion potentials.[6] This means, that sufficient ionic contact is mandatory for a correct measurement of the electrode potentials. If the contact is insufficient, the potential of the side reaction is measured instead.

Figure S17d and e show how the potential at the reference electrode is only affected by the side reaction, if the conductivity is sufficiently low, thus providing the explanation to the observations in Figure S17b and c.

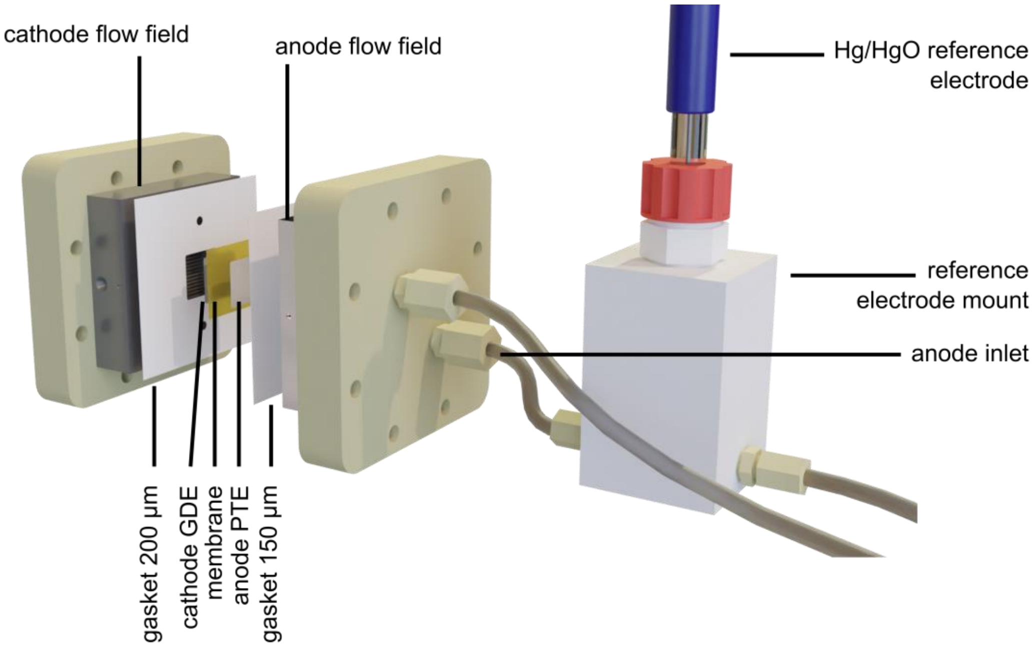

Figure S20: Electrolyte reference electrode setup. Setup scheme with utilized gaskets and housing for the reference electrode.

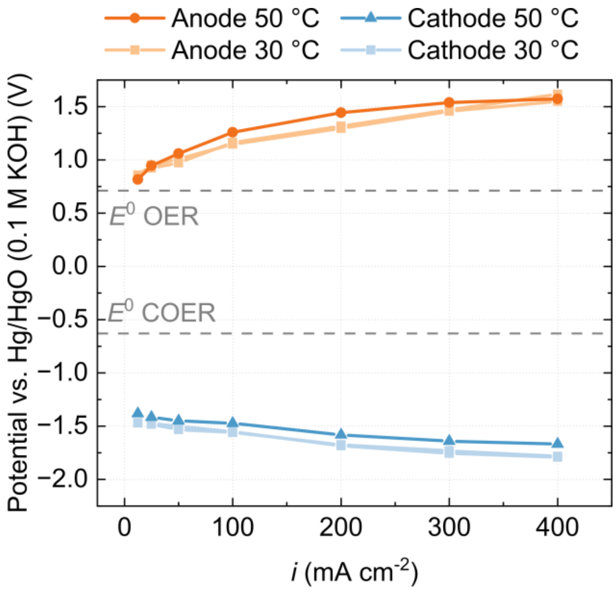

Figure S21: Comparison of electrode potentials measured in edge-type reference electrode setup at 50 °C and 30 °C.

References

1 W.H. Lee, Y.-J. Ko, Y. Choi, S.Y. Lee, C.H. Choi, Y.J. Hwang, B.K. Min, P. Strasser, H.-S. Oh, *Nano Energy*, **2020**, 105030.

2 Y. Zhao, N.M. Vargas-Barbosa, E.A. Hernandez-Pagan, T.E. Mallouk, *Small (Weinheim an der Bergstrasse, Germany)*, **2011**, *14*, 2087.

3 L.V. Bühre, S. Bullerdiek, P. Trinke, B. Bensmann, A.-L.E.R. Deutsch, P. Behrens, R. Hanke-Rauschenbach, *J. Electrochem. Soc.*, **2022**, *12*, 124513.

4 Inzelt, G., Lewenstam, A., Scholz, F., Baucke, F.G.K., *Handbook of reference electrodes*, Springer, Berlin, New York, **2013**.

5 Kieninger, J., *Electrochemical Methods for the Micro- and Nanoscale*, De Gruyter, **2022**.

6 H. Becker, L. Castanheira, G. Hinds, *Journal of Power Sources*, **2020**, 227563.
